# An Optimally Regularized Estimator of Multilevel Latent Variable Models with Improved MSE Performance

**DOI:** 10.1017/psy.2025.10045

**Published:** 2025-09-22

**Authors:** Valerii Dashuk, Martin Hecht, Oliver Lüdtke, Alexander Robitzsch, Steffen Zitzmann

**Affiliations:** 1 Hector Research Institute of Education Sciences and Psychology, University of Tübingen, Germany; 2 Department of Psychology, Faculty of Human Sciences, MSH Medical School Hamburg, Germany; 3 Department of Methodology and Statistics for Psychology, Helmut Schmidt University Hamburg, Germany; 4 Educational Measurement and Data Science, Leibniz Institute for Science and Mathematics Education at the University of Kiel, Germany; 5 Educational Measurement and Data Science, Centre for International Student Assessment, Germany

**Keywords:** intraclass correlation, mean squared error, multilevel latent variable model, regularized estimation, small sample

## Abstract

We propose an optimally regularized Bayesian estimator of multilevel latent variable models that aims to outperform traditional maximum likelihood (ML) estimation in mean squared error (MSE) performance. We focus on the between-group slope in a two-level model with a latent covariate. Our estimator combines prior information with data-driven insights for optimal parameter estimation. We present a “proof of concept” by computer simulations, involving varying numbers of groups, group sizes, and intraclass correlations (ICCs), which we conducted to compare the newly proposed estimator with ML. Additionally, we provide a step-by-step tutorial on applying the regularized Bayesian estimator to real-world data using our MultiLevelOptimalBayes package.

Encouragingly, our results show that our estimator offers improved MSE performance, especially in small samples with low ICCs. These findings suggest that the estimator can be an effective means for enhancing estimation accuracy.

## 1 Introduction

Multilevel latent variable models have been widely adopted in psychology, education, and related sciences to analyze hierarchical data while accounting for unobserved effects (Bollen et al., 2022; Lüdtke et al., 2008; Skrondal & Rabe-Hesketh, 2009; Zitzmann, Wagner, et al., 2022). Unlike traditional multilevel regression models (Raudenbush & Bryk, 2002; Snijders & Bosker, 2012), which rely on observed variables at each level, multilevel latent variable models introduce latent constructs that improve measurement accuracy and reduce bias in parameter estimates (Muthén & Asparouhov, 2012; Zitzmann et al., 2016). These models allow for more precise estimations of relationships at different levels of analysis by correcting for measurement error and providing a more flexible framework for capturing complex dependencies in nested data.

Over the past two decades, multilevel latent variable models have been widely applied in educational research to model student achievement and classroom effects (Lüdtke et al., 2008; Marsh, 1987), psychological research for latent personality and cognitive processes (Bollen et al., 2022; Muthén & Asparouhov, 2012), and health sciences for hierarchical patient-reported outcomes (Hamaker & Klugkist, 2011).

Compared to mixed-effects models (Raudenbush & Bryk, 2002; Snijders & Bosker, 2012), which typically assume that all predictors are observed and measured without error, multilevel latent variable models provide greater flexibility in handling measurement error and latent constructs. This makes them particularly valuable in psychological and educational research, where many key variables (e.g., cognitive ability, motivation, and instructional quality) cannot be directly observed. Moreover, multilevel latent variable models allow researchers to separate within-group and between-group variance more effectively than traditional mixed-effects models, leading to more reliable inferences.

Multilevel models can be classified based on whether variables are assessed at the individual or group level (Croon & van Veldhoven, 2007; Snijders & Bosker, 2012). One relevant example in education is the study of student learning outcomes as a function of class-level characteristics such as class size. The “classic” multilevel models (also called random intercept models) used for this purpose are often estimated using software, such as HLM (Raudenbush et al., 2011) or lme4 (Bates et al., 2015).

However, various works (e.g., Asparouhov & Muthén, 2007; Lüdtke et al., 2008) have argued that this type of aggregation can lead to severely biased estimates of the effect of the context characteristic. One possible solution is to use a specialized multilevel model in which the context variable is formed through latent rather than manifest aggregation (for a discussion of latent aggregation, see Lüdtke et al., 2008, 2011). Unfortunately, such a model with a latent predictor cannot be specified in HLM or lme4 and is therefore often estimated using Mplus (Muthén & Muthén, 2012). However, these models place high demands on the data, and convergence problems or inaccurate estimates of effects at the class level (accuracy issues) can occur.

Similar methods also play a role in other modeling contexts, such as regression analysis (Hoerl & Kennard, 1970; Tibshirani, 1996; see also McNeish, 2015) and structural equation models (Yuan & Chan, 2008; see also Yuan & Chan, 2016). In the latter, a small value is typically added to the estimated variance, and it has been suggested that a similar effect can be achieved by selecting an appropriate prior distribution (e.g., Chung et al., 2015; McNeish, 2016; Zitzmann et al., 2016).

Bayesian approaches have gained increasing popularity in multilevel modeling due to their ability to enhance estimation accuracy by incorporating prior information (Hamaker & Klugkist, 2011; Lüdtke et al., 2013; Muthén & Asparouhov, 2012; Zitzmann et al., 2015, 2016). The possibility of adding prior information is a fundamental aspect of Bayesian estimation. It combines information from the data at hand, captured by the likelihood function, with additional information from prior distribution, resulting in inferences based on the posterior distribution (Gelman, 2006). However, specifying priors can pose challenges, particularly in small samples with a low intraclass correlation (ICC), where the choice of prior is crucial (Hox et al., 2012). Small sample sizes are very common in psychology and related sciences due to limitations in funding and resource constraints (Browne & Draper, 2006). In such cases, between-group estimates may approach zero and become unstable, significantly increasing sensitivity to prior specification. This makes prior misspecification one of the biggest challenges in applying Bayesian approaches to latent variable models (Natarajan & Kass, 2000; Zitzmann et al., 2015). However, this effect of the prior can also be exploited. Recent research by Smid et al. (2020) has shed light on the importance of constructing “thoughtful priors” based on previous knowledge to enhance estimation accuracy (see also Zitzmann, Lüdtke, et al., 2021). In the Bayesian approach proposed in this article, the prior parameters are determined through a theoretically derived automated procedure that minimizes the estimated mean squared error (MSE). This removes the need for the user to manually specify a prior, thereby eliminating the risk of user-induced misspecification.

While Smid et al. (2020) focused on addressing small-sample bias, it has been argued that evaluating the quality of a method should consider not only bias but also the variability of the estimator, particularly in small samples with low ICCs (Greenland, 2000; Zitzmann, Lüdtke, et al., 2021). In cases of low ICCs, within-group variability dominates, and small sample sizes lead to unstable group-level estimates, resulting in higher variance when estimating between-group slopes. This highlights a crucial point—approaches solely dedicated to minimizing bias may, in fact, perform less optimally than those focused on reducing variability alone. Thus, it is important to consider both bias and variability in optimizing analytical strategies. In this regard, alternative suggestions for specifying priors have aimed at reducing the MSE, which combines both bias and variability (e.g., Zitzmann et al., 2015, 2016). Note that in cases of small samples and low ICCs, MSE is largely driven by the variability of the estimator. Therefore, minimizing variability remains an important goal when optimizing MSE.

In the same spirit, in this article, we derive a distribution for the Bayesian estimator of between-group slopes, building on the model originally established by Lüdtke et al. (2008). Specifically, we use this distribution to develop an optimally regularized Bayesian estimator that automatically selects priors to minimize MSE, thereby avoiding misspecification caused by user-specified priors. We then report the results from computational simulations conducted across a broad spectrum of conditions to evaluate the estimator. They demonstrate the advantages of this approach compared to ML estimation, particularly in scenarios of small samples and a low ICC.

## 2 Theoretical derivation

Before delving into detailed aspects, we will briefly summarize Lüdtke et al.’s (2008) model, which we use to exemplify our approach. This model was proposed as one way to provide unbiased estimates of between-group slopes in contextual studies. It proposes predicting the dependent variable *Y* at the group level by using a latent variable. This latent variable represents a group’s latent mean, offering a more reliable alternative than the traditional manifest mean approach. Known as the “multilevel latent covariate model,” this model allows for the integration of latent group means into the more complex frameworks of multilevel structural equation models, which are prevalent in psychological research and related research (see also Zitzmann, Lohmann et al., 2022).

Zitzmann, Lüdtke, et al. (2021) have proposed and discussed a Bayesian estimator for the between-group slope in this model (see also Zitzmann & Helm, 2021). Their approach introduced a method for incorporating prior information in estimating between-group slopes. However, this method required manual specification of prior distributions, which could be challenging, particularly in small samples where misspecified priors may lead to biased or unstable estimates. In contrast, our approach extends this work by upgrading their Bayesian estimator to a regularized Bayesian estimator that automatically selects optimal priors, thereby preventing user misspecification and improving estimation stability.

Since our method regularizes the estimator introduced by Zitzmann, Lüdtke, et al. (2021), we maintain their notation for consistency. More precisely, in the model, it is assumed that the individual-level predictor *X* is decomposed into two independent, normally distributed components: 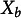 , representing the latent group mean, and 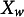 , representing individual deviations from 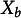 . Thus, for an individual 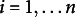 within a group 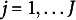 , the decomposition can be stated: 
(1)

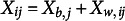



(2)

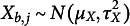



(3)

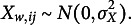

Note that further, we assume that each of *J* groups includes *n* persons, therefore the overall sample size is 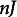 .

Hereafter, we will refer to 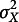 and 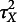 as the within-group and between-group variances of *X*, respectively. Similarly, 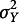 and 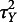 are the within-group and between-group variances of *Y*, respectively.

The individual-level and group-level regressions read: 
(4)

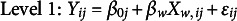



(5)

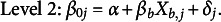

In Equation (4), 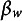 represents the within-group slope that characterizes the relationship between the predictor and the dependent variable at the individual level, while 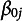 describes the random intercept. Normally distributed residuals are denoted as 
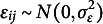
 .

**Figure 1:**
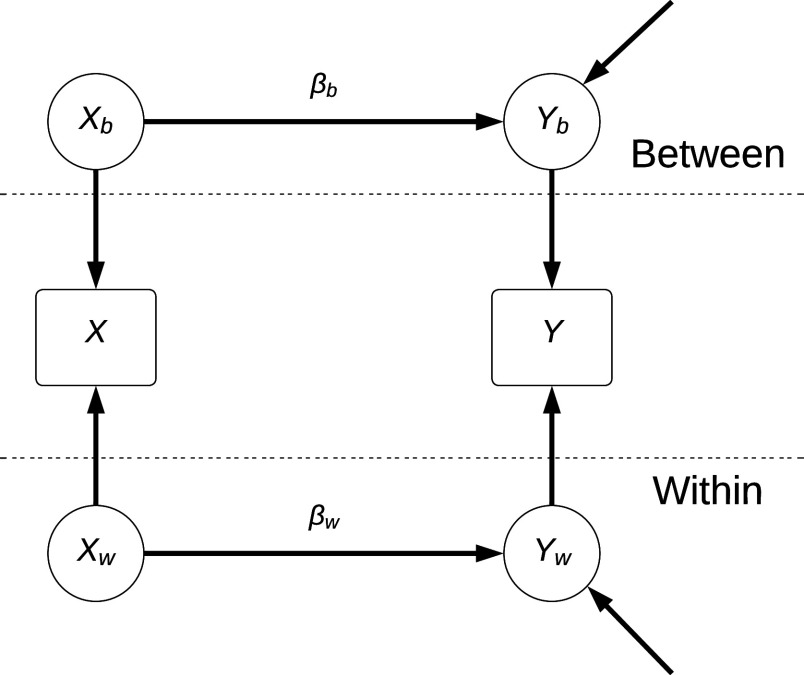
A multilevel structural equation model using the within-between framework that decomposes the variables *X* and *Y* into within-group and between-group components. *Note*: The within-group components are denoted by subscript *w*, and the between-group components are denoted by subscript *b*. The between-group components ( 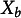 and 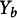 ) are connected through a regression, where 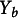 serves as the dependent variable and 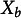 as the predictor. Similarly, the within-group components ( 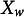 and 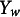 ) are related to each other in an analogous manner. The notation includes 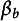 for the between-group slope and 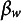 for the within-group slope.

Moreover, we denote between-group slope in Equation (5) as 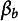 and the overall intercept as 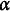 . 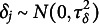 represents normally distributed residuals. See Figure 1 for a visual representation of the model. Note that the between-group component 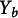 in Figure 1 corresponds to the random intercept 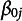 in Equation (4), whereas the within-group component 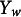 in Figure 1 corresponds to 
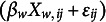
 in Equation (4).

We focus on the between-group slope 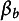 , which is the most important parameter in numerous multilevel model applications, such as when analyzing contextual effects. For balanced data (where each group has an equal number of individuals), the maximum likelihood (ML) estimator of 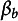 is given by: 
(6)

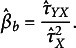

In this equation, 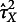 and 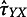 are sample estimators of the group-level variance of *X* and the group-level covariance between *X* and *Y*, respectively.

While the asymptotic properties of the ML estimator (6) are advantageous, it tends to exhibit bias in finite sample sizes and displays significant variability, leading to a substantial MSE in such scenarios (as demonstrated by, e.g., McNeish (2016)). This poses a challenge to the practical utility of the ML estimator for rather small samples with low ICCs, as results from individual studies could be notably imprecise. Consequently, researchers have recommended alternative estimators that demonstrate lower variability, leading to increased accuracy and a reduced MSE, although potentially at the cost of some more bias compared to the ML estimator. Notable among these are the estimators proposed by Chung et al. (2013), Zitzmann et al. (2015), and Zitzmann, Lüdtke, et al. (2021); see also Zitzmann & Helm (2021). Next, we will develop a regularized version of Zitzmann, Lüdtke, et al.’s Bayesian estimator for the between-group slope, drawing on the so-called indirect strategy approach of constructing the estimator outlined by Zitzmann, Lüdtke, et al. (2021). The details of this development are provided in Appendix A.

Zitzmann, Lüdtke, et al.’s (2021) Bayesian estimator starts with the prior gamma distribution and its two parameters, 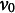 and 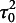 (see Appendix A). A specific choice of prior parameters is not required, as our forthcoming Bayesian estimator is designed to find the optimal values to minimize MSE. Combining priors with the ML estimator, Zitzmann, Lüdtke, et al. (2021) derived the Bayesian estimator as 
(7)

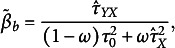

where 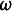 is the weighting parameter defined as a function of the gamma-distributed priors. The denominator in Equation (7) accounts for both the prior variance 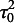 and the observed between-group variance 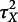 , with weights adjusted by 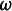 to control the influence of prior information as *J* increases.

Practically, 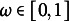 can be interpreted as the relative weight given to the prior versus the data-based estimate: 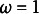 corresponds to the standard ML estimator (Equation (6)), 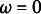 corresponds to full shrinkage toward the prior mean, and intermediate values balance the two sources of information.

The derivation of the Bayesian estimator (Equation (7)) is described in detail in Appendix A. Note that Equation (7) is essentially a Stein-type estimator (Stein, 1956).

We specify the weighting parameter (prior) 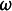 in a manner similar to that of Zitzmann, Lüdtke, et al. (2021): 
(8)

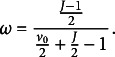



So 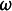 is defined as a function of the gamma-distributed prior 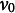 and the number of groups *J*. The weighting factor 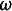 is derived such that as 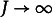 , 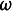 approaches 1, ensuring that the Bayesian estimator converges to the ML estimator. Note that the weighting parameter 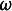 in Equation (8) differs from the one introduced by Zitzmann, Helm, and Hecht (2021) because we further optimize it (see Appendix A).^1^

The Bayesian estimator 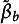 is not yet regularized. To this end, the two parameters 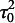 and 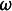 need to be identified. As mentioned, 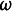 is defined as a function of sample size and converges to 1 when 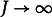 . Therefore, the Bayesian estimator 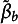 is asymptotically unbiased and coincides with the ML estimator 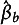 in Equation (6) when samples are sufficiently large. In finite samples, however, the Bayesian estimator is biased.

To obtain the optimally regularized 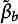 , it is essential to find the values for 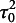 and 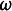 based on an optimality criterion. The MSE serves as the natural choice for this criterion. It is defined as 
(9)

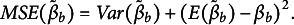

As can be seen from the equation, this measure is simply the sum of the variance and the squared bias of the estimator. As the ML estimator in Equation (6) is unbiased in theory, its MSE shortens just to the variance of this estimator. The Bayesian estimator as defined in Equation (7) does not share the same unbiasedness property. Rather, it reduces the MSE by reducing its variance at the cost of some bias. We will show how to construct the estimator in such a way that a substantially reduced MSE is achieved compared to the ML estimator 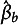 in small samples with low ICCs. In infinite samples, the MSE of 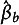 reaches its global minimum of 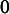 (as both variance and bias converge to 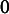 ), and due to the weighting parameter 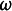 , the Bayesian estimator 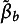 achieves the same outcome.

To find the optimal values of the parameters 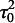 and 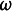 , it is necessary to express the between-group (co)variance estimators from Equation (7), 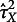 and 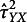 , in terms of the normal distributions of the between- and within-group components of the predictor and the dependent variable, namely, 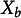 , 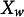 , 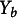 , and 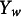 (see Appendix B for more details). We derived the expression for 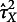 under the restriction that it should have an easily definable distribution. For the derivation, see Appendix B. This resulted in 
(10)

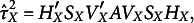

where 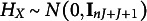 . The coefficient matrix *A* is defined in Equation (F.1) of Appendix F. Additionally, matrices 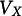 and 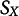 are the matrices of eigenvectors and eigenvalues, respectively. They are defined in Equation (B.30) of Appendix B. The internal part of Equation (10), 
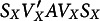
 , is a diagonal coefficient matrix. This means that in Equation (10), we express 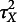 as a weighted sum of squares of independent normally distributed random variables, that is, a weighted sum of 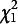 -distributed random variables, which are transformed from 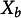 , 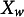 , 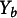 , and 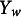 .

To express 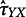 , we use a similar transformation as for 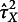 . This transformation is described in detail in Appendix C. The result is 
(11)

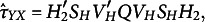

where 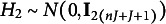 is a multivariate standard normally distributed random vector. Coefficient matrix *Q* is computed in Equation (C.15) of Appendix C. Matrices 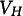 and 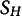 are the matrices of eigenvectors and eigenvalues, respectively. They are defined in Equation (C.12) of Appendix C. Furthermore, the internal part of Equation (11), 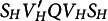 , is a diagonal coefficient matrix. With Equation (11), the estimator of the group-level covariance 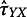 is represented as a weighted sum of squares of independent normally distributed random variables, that is, a weighted sum of 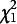 -distributed random variables.

As a consequence, we express each of the estimators of group-level (co)variances 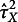 and 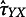 as a sum of squares of independent and identically distributed normal random variables in Equations (10) and (11), respectively. Every term of these sums is 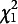 -distributed, thus following the Gamma 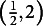 distribution. Notice that a gamma distribution can be scaled: if a variable 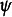 follows the Gamma 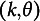 distribution, then 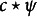 is Gamma 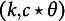 -distributed. Therefore, we can represent the estimators of group covariances, 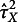 and 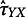 , as gamma-distributed random variables: 
(12)

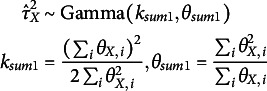



(13)

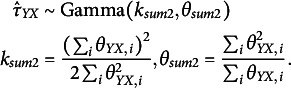

The scales 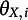 and 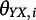 are the elements of the diagonal matrices 
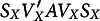
 (for 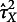 ) and 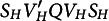 (for 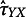 ) in Equations (10) and (11).

In the next step, we make use of the distributions of the sample covariances 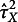 and 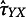 to calculate the distributions of the ML estimator 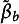 and the Bayesian estimator 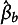 . The estimators 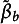 and 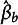 are defined using an *F* distribution, because ratios of gamma-distributed random variables follow *F* distributions. The full procedures of deriving the distributions of 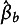 and 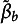 are presented in Appendix D. The results of these derivations are the following distributions: 
(14)

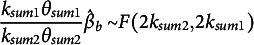



(15)

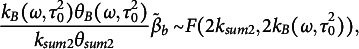

where the coefficients 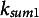 , 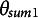 , 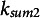 , 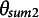 , 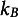 , and 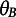 are defined and fully described in Equations (D.3), (D.4), (D.9), and (D.10) of Appendix D. Note that 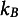 and 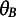 are functions of the prior parameters 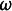 and 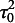 . Using these distributions, we compute the variances and expected values of both estimators and combine them into the final formulas for their MSEs: 
(16)

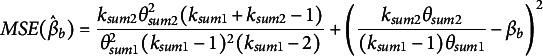



(17)

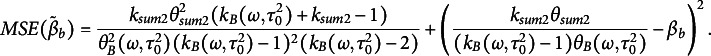



As a byproduct, we obtain their standard errors from the estimators’ distributions as 
(18)

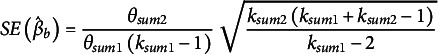



(19)

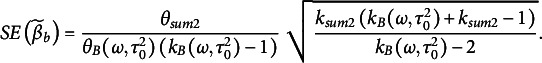



Using these standard errors, one can describe the uncertainty associated with the estimation or use them for statistical testing. However, when samples are rather small, we recommend to use resampling procedures for obtaining standard errors, such as the delete-d jackknife (Shao & Wu, 1989; for applications in multilevel modeling, see Zitzmann, 2018; Zitzmann et al., 2022; Zitzmann et al., 2023, 2024).

Having obtained the MSE of 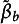 (Equation (17)), we can minimize it with respect to the parameters 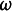 and 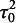 in order to obtain our regularized Bayesian estimator. To find the optimal choices for the prior parameters, we employ a numerical approach, which is algorithmic in nature, making it well-suited for implementation in software platforms like R or MATLAB. The algorithm is a grid search over the parameters, with 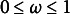 and 
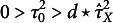
 . Since it is impossible to find the global minimum in the general case (Lakshmanan, 2019), the algorithm we implement performs only a local optimum search. We propose to choose parameter *d* to be at least five times the standard deviation of the estimated group-level variance of *X*, that is, 
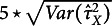
 . The value of 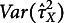 may be obtained from the derived distribution of 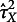 in Equation (D.3) of Appendix D, or even more exactly, by using the procedures of Mathai (1993) or Fateev et al. (2016). This 5-sigma region guarantees that the minimum estimated MSE falls inside this region with high probability. The probability of the minimum estimated MSE being within this interval is at least 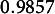 for 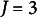 , 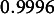 for 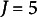 , and 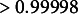 for 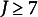 . In this case, our grid search will find the inner solution for the optimal values of 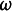 and 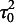 that minimize the estimated MSE. Note that the grid search algorithm minimizes the estimated MSE but not the unknown true MSE.

It is important to note that the MSE in Equations (16) and (17) incorporates the unknown between-group coefficient 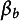 . We propose using its ML estimate, 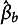 , as a substitute, thereby giving our technique an empirical Bayes flavor. Such uses of “plug-in estimates” are not uncommon in statistics and often very useful (Liang & Tsou, 1992; see also Zitzmann et al., 2024).

We have demonstrated an approach for minimizing the MSE of the between-group parameter, leading to what we refer to as the optimally regularized Bayesian estimator 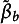 for this parameter. Notice that our estimator uses the ML estimator 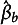 during MSE optimization and even includes ML as a special case when 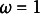 . This means, in small samples, we can do better than the ML estimator in terms of MSE. However, when working with large sample sizes, the costs due to using approximate distributions and the plug-in procedure to compute the regularized Bayesian estimator may be larger than the benefits. Such a scenario is likely to occur with larger group sample sizes combined with high levels of the ICC of the predictor. In the next section, we demonstrate some of these properties using simulated data.

## 3 Simulation studies

We begin with the description of the data-generating mechanism, including its parameters, such as group size *n*, number of groups *J*, ICC coefficient ICC 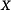 , and the coefficients 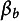 and 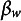 . We utilized the generated data to compute estimates using both the proposed optimally regularized Bayesian estimator and, for benchmarking purposes, also the ML estimator. The full algorithm used to actually yield 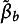 is detailed in Appendix E. Finally, we present the results graphically. Detailed results can be found in Appendix G, which allows for a more comprehensive evaluation of the estimation accuracy under varying input parameters.

### 3.1 Data generation

Next, we detail the data generation process and outline the specifics of our simulation setup. We base our simulations on the data-generating process used by Zitzmann, Helm, and Hecht (2021) and Zitzmann, Lüdtke, et al. (2021). Specifically, we conducted simulations for each unique combination of the following parameters:

- ICC 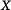 : Intraclass Correlation (0.05, 0.1, 0.3, 0.5).
- *J*: Number of groups (5, 10, 20, 30, 40).
- *n*: Number of individuals per group (5, 15, 30).

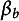
 : Between-group parameter (0.2, 0.5, 0.6).

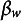
 : Within-group parameter (0.2, 0.5, 0.7).

In total, this resulted in 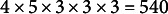 scenarios, each of which was replicated 5,000 times. The relatively small number of groups was chosen to reflect reasonable two-level scenarios in the social sciences (i.e., typically 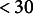 students per class, 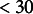 schools per district), and to align with examples from Gelman & Hill (2006).

The values of 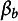 and 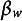 follow ranges used in prior simulation studies on the multilevel latent covariate framework and related models. For example, Lüdtke et al. (2008) used values 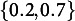 , Grilli & Rampichini (2011) considered values including 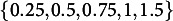 , and Zitzmann & Helm (2021) used the value of 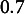 . The combination 
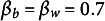
 is infeasible under our fixed ICC 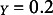 design, so 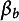 was reduced to 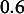 in that case. Similarly, near-zero 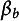 values were not included because for ICC 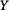 = 0.2, they would violate ICC constraints: 
(20)

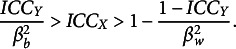

The ICC of the dependent variable, denoted as ICC 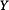 , was preset to 0.2 within the code to study scenarios with ICC values that lie at the center of the typical ICC range observed in empirical studies (Gulliford et al., 1999). Additionally, we incorporated another validity check in order to identify and exclude incorrectly specified inputs, such as non-integer values for *J* or *n*.

### 3.2 Evaluation criteria

The goal of our simulations was to assess how well the regularized Bayesian estimator can estimate the true parameter value 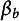 across various scenarios. To this end, we assessed its performance in terms of the MSE and bias. Note that in addition to the presented estimator, a variant thereof was studied. Both variants were compared against the ML estimator.

We consider the following variants of the regularized Bayesian estimator: our proposed Bayesian estimator with the MSE optimization based on plugged-in ML-estimate 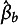 ; Bayesian estimator with MSE optimization based on the true value of 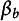 .

It is important to note that only the variant-1 Bayesian estimator (with MSE optimization based on the ML estimate 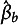 ) and the ML estimator are practically applicable to real data. In contrast, the second Bayesian estimator (with MSE optimization based on the true 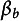 ) serves only as a theoretical benchmark.

Further, as evaluation measures, we use the square root of the MSE, denoted as RMSE, and the relative bias. First, MSE is computed as the mean of the squared differences between the estimated parameter and the true between-group parameter, 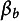 . Second, the square root is taken to obtain RMSE from MSE. RMSE then allows for comparisons similar to those made with MSE^2^ while presenting the error in the original units of measurement. Our preference for RMSE over MSE stems from its scalability and straightforward interpretability. These attributes enhance the visualization of our analysis, facilitating clearer insights into the estimators’ performance. The RMSE describes the overall accuracy of parameter estimation, indicating the proximity of estimated values to the true parameter values. Relative bias, in contrast, assesses the average deviation of the estimated parameters from the true value. It is computed as the ratio of the mean difference between the estimated parameter and the true between-group parameter to the true between-group parameter, 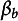 . The mean difference is calculated over repeated replications of each scenario in our simulation study. A small relative bias indicates that the estimator produces results that, on average, are closer to the true parameter value, while a larger relative bias suggests systematic overestimation or underestimation.

### 3.3 Simulation results

**Figure 2:**
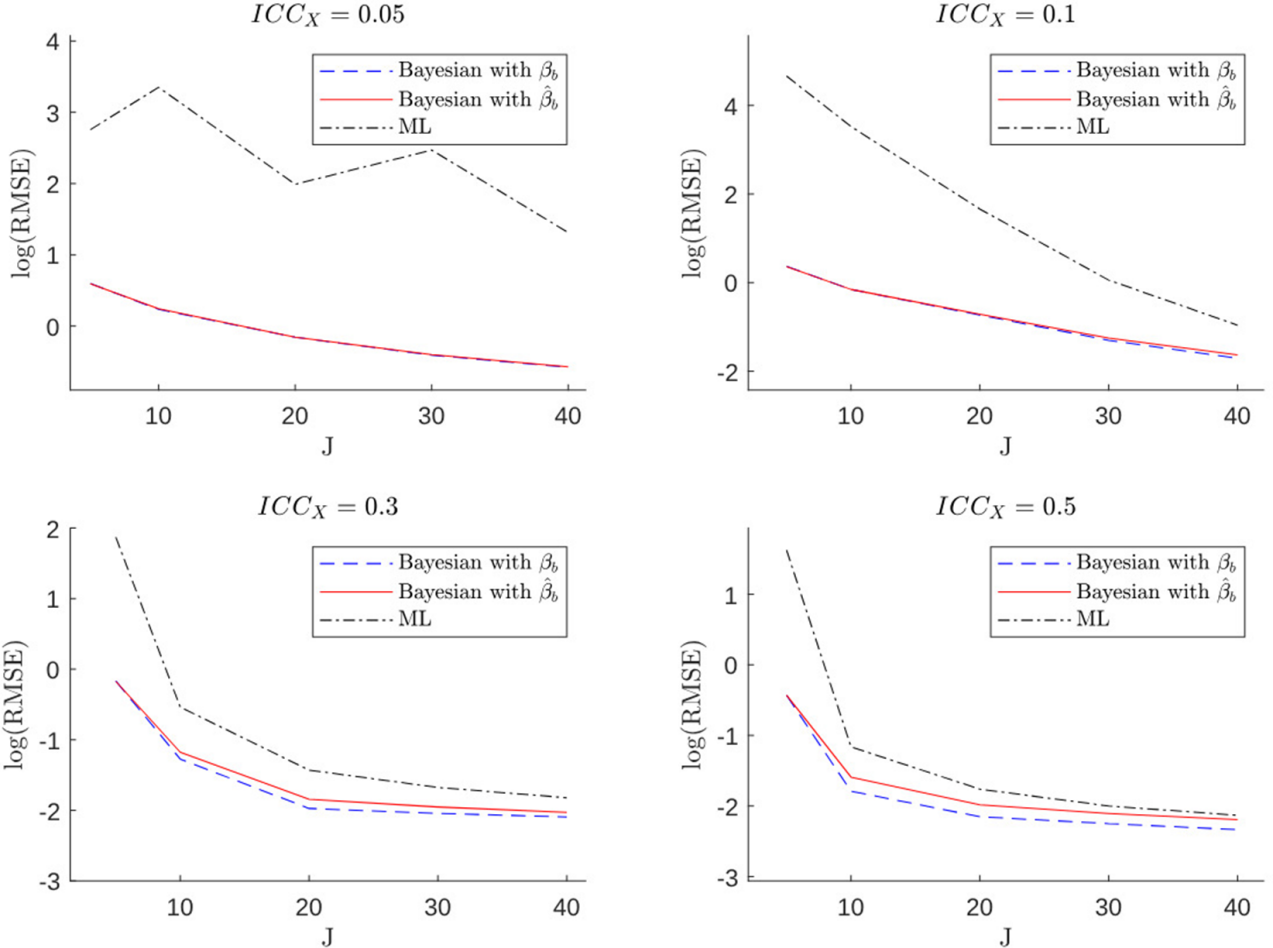
Log of root MSE (RMSE) in estimating the between-group slope 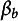 for the ML and the two Bayesian estimators as a function of the sample size at the group level (*J*) and the ICC of the predictor ICC 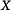 . *Note*: The scale of the *y*-axis differs between the four subplots. Results are shown for 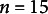 people per group, and constant within-group and between-group slopes of 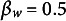 and 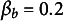 , respectively.

**Figure 3:**
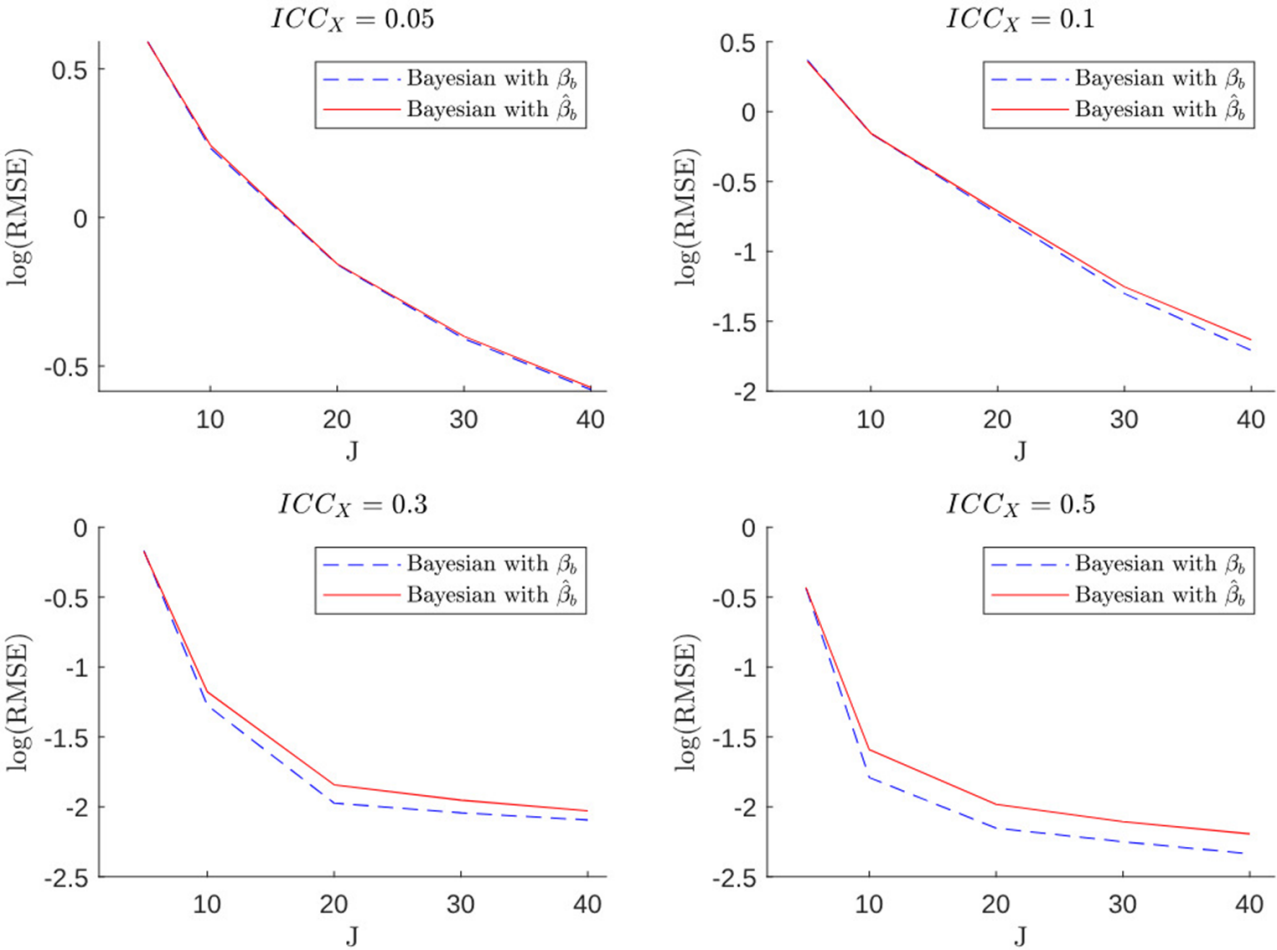
Log of root MSE (RMSE) in estimating the between-group slope 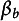 for the two Bayesian estimators as a function of the sample size at the group level (*J*) and the ICC of the predictor ICC 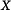 . *Note*: The scale of the *y*-axis differs between the four subplots. Results are shown for 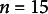 people per group, and constant within-group and between-group slopes of 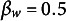 and 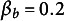 , respectively.

**Figure 4:**
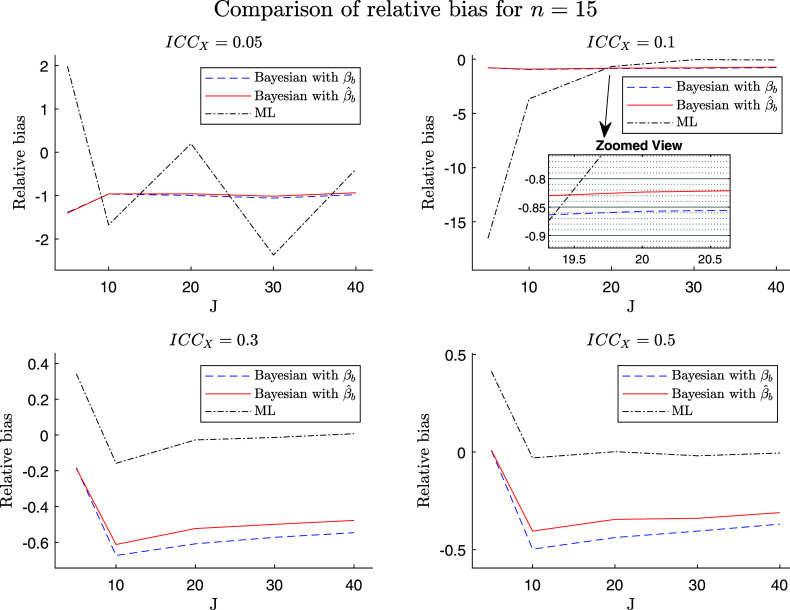
Relative bias in estimating the between-group slope 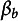 for the ML and the two Bayesian estimators as a function of the sample size at the group level (*J*) and the ICC of the predictor ICC 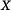 . *Note*: The scale of the *y*-axis differs between the four subplots. Results are shown for 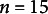 people per group, and constant within- and between-group slopes of 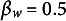 and 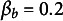 , respectively.

Here, we report the results of our simulation study, focusing on the characteristics of the simulated data, their alignment with theoretical expectations, and the comparisons between our proposed estimator, the variant thereof, and the ML estimator. To facilitate a better understanding, we present visual analyses in Figures 2–4, which illustrate the differential behaviors of the estimators as a function of the group-level sample size and the ICC. For a better differentiation between methods, we chose to show the logged RMSE in Figures 2 and 3. Note that log is a monotone increasing function for RMSE 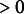 .

For more details about the RMSE and relative bias across 540 unique scenarios, see Tables 2–9 (see Appendix G).

Figure 2 provides a visual representation of the log of the RMSE patterns for the three estimators of the slope. The first line (blue dashed line) in Figure 2 is from the second alternative variant of the Bayesian estimator; that is, the Bayesian estimator based on the true value of 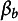 and thus the direct implementation of Equation (17). As mentioned, this estimator cannot be used on the real data, as the 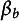 is unknown, but it works as a benchmark for comparison with our proposed Bayesian estimator. This latter estimator (red solid line) is the Bayesian estimator with the plug-in ML estimate 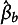 in place of 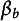 . The third estimator (black dash-dot line) is the ML estimator. Recall that among the three estimators, only the second and third are applicable to the real data.

Our theoretical expectations align with the observed trends, as both Bayesian estimators exhibit lower RMSE compared to the ML estimator. This RMSE reduction is more pronounced for smaller group sizes (*J*), with the effect amplified by lower intraclass correlations (ICC 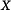 ). Additionally, RMSE consistently decreases with increasing *J* for all methods and ICC levels. However, an exception is observed for the ML estimator in the upper left plot of Figure 2, where RMSE does not follow this expected trend. At low ICC 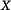 and small *J*, between-group variance 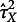 is often estimated near zero, causing the ML estimator (Equation (6)) to inflate and produce occasional extreme values. This yields a finite-sample distribution that mixes regular estimates with such extremes. Because RMSE is highly sensitive to these rare events, the population RMSE can display non-monotonic patterns across adjacent *J* values even with very large numbers of replications. In contrast, the regularized Bayesian estimators replace 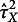 with 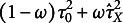 in the denominator, bounding it away from zero and producing smooth, strictly decreasing RMSE curves. Despite this, the overall comparison remains valid, as ML consistently underperforms the regularized Bayesian estimators across all analyzed scenarios in Figure 2.

Figure 3 further adds to the understanding of the performance differences. This figure demonstrates that the differences in RMSE between Bayesian estimators based on inserting the true versus estimated values of 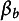 are only negligible, speaking for the usefulness of the Bayesian estimator with the plugged-in ML estimate of 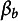 .

Figure 4 shows the behavior of the estimators with respect to the relative bias. The first thing to mention is that both variants of the Bayesian estimator (blue dashed and red solid lines) do not converge to a bias of zero with an increasing, but finite number of groups *J*, while the ML estimator does (black dash-dot line). This bias is not due to misspecified priors but is the intended result of MSE-optimal shrinkage in the Bayesian estimator (Equation (7)), where bias is deliberately traded for reduced variability. However, as 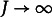 and 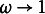 , the regularized Bayesian estimator converges to ML, and the bias disappears. Secondly, with an increasing intraclass correlation ICC 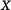 , the relative bias of all three estimators decreases (plots 1–4 of Figure 4). Thirdly, despite being asymptotically unbiased, the ML estimator exhibits small-sample bias, especially for small ICC values (see upper left plot in Figure 4). This bias is inherent to ML estimation and results from denominator instabilities when 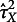 (Equation (6)) is estimated near zero under low ICC, which can lead to sporadic extreme values and a heavy-tailed error distribution. This effect occurs only with the ML estimator, whereas the regularized Bayesian approaches remain stable across all scenarios because the denominator uses the weighted sum 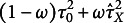 (Equation (7)).

Table 1 presents RMSE and relative bias values computed across all 540 scenarios and averaged within each combination of group size *n* and number of groups *J*. It consolidates information from Tables 2–9 in Appendix G. Specifically, Table 1 compares three estimators: maximum likelihood (ML), regularized Bayesian with 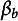 , and regularized Bayesian with 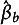 . Highlighted cells identify the estimator with the smallest RMSE (and therefore the smallest MSE) and the smallest relative bias. Results clearly illustrate that, across all examined cases, the regularized Bayesian estimators consistently provide lower RMSE values compared to the ML approach. However, as both group size and the number of groups increase, the relative bias of the ML estimator approaches zero, as it is a consistent estimator. At the same time, the relative bias of the regularized Bayesian estimators remains around 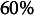 . Consequently, for larger *n*, the ML estimator often has the smallest highlighted relative bias. Nevertheless, even when the ML estimator exhibits less bias than both regularized Bayesian estimators, the regularized Bayesian estimators achieve a substantial reduction in MSE and RMSE values, especially when *n* and *J* are small. Thus, Table 1 emphasizes that, according to our simulation studies, regularized Bayesian estimation—where only the regularized Bayesian estimator with 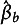 is applicable in the real world—may deliver more biased estimations, compared to ML, but is highly preferable in terms of MSE, especially in scenarios with small *n* and *J*.

**Table 1:** Average RMSE and relative bias values of the ML (RMSE 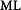 and Bias 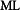 , respectively), the Bayesian estimator with 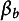 (RMSE 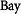 and Bias 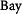 , respectively), and the Bayesian estimator with 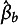 (RMSE 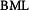 and Bias 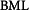 , respectively) for different values of *n* and *J*. Values in bold indicate the smallest RMSE and the smallest relative bias for each combination of *n* and *J*

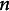	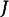	RMSE 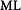	RMSE 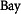	RMSE 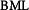	Bias 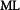	Bias 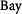	Bias 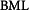
5	5	138.948	2.165	**2.139**	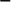 541.286	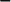 85.861	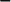 **85.565**
5	10	65.035	**1.230**	1.231	**20.007**	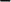 79.511	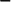 77.130
5	20	101.584	**0.771**	0.781	253.325	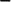 67.544	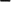 **64.531**
5	30	20.412	**0.602**	0.611	**33.315**	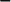 59.854	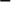 56.847
5	40	25.685	**0.519**	0.526	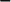 60.882	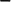 57.754	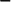 **54.792**
15	5	456.334	1.131	**1.129**	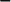 2815.721	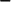 31.872	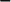 **31.855**
15	10	107.527	**0.653**	0.662	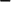 564.371	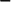 51.219	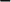 **48.227**
15	20	19.847	**0.443**	0.451	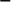 79.606	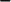 54.971	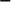 **51.664**
15	30	7.720	**0.362**	0.368	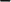 **5.551**	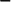 55.591	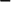 52.659
15	40	3.561	**0.315**	0.320	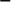 **4.161**	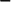 55.796	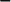 53.163
30	5	84.649	**0.949**	0.950	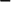 88.566	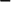 20.531	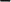 **20.521**
30	10	19.940	**0.546**	0.556	**16.845**	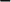 52.950	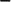 49.524
30	20	4.110	**0.341**	0.347	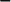 **12.779**	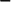 57.784	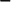 54.571
30	30	0.473	**0.279**	0.283	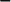 **1.565**	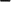 57.588	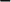 54.760
30	40	0.386	**0.257**	0.260	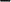 **1.737**	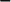 56.888	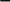 54.412

In conclusion, our optimally regularized Bayesian estimator with the ML estimate plugged in demonstrates its power to refine the accuracy of estimators for the between-group slope 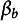 in small samples. While acknowledging inherent bias (see Table 3 in Appendix G for details), this estimator generated through our approach demonstrates enhanced accuracy when juxtaposed with the ML estimator, particularly in situations characterized by a finite sample size. Next, we provide a summary of our introduced approach, reflect on the theoretical advancements, highlight new findings, address limitations, and offer insights into the broader implications of our work.

## 4 Step-by-step tutorial using MLOB R package

To illustrate the practical application of the newly developed estimator, we created the MultiLevelOptimalBayes (MLOB) package, which includes the estimation function mlob(). In this section, we provide step-by-step instructions on using the regularized Bayesian estimator with the MLOB package in R. The estimator is applied to the PASSNYC dataset—a real-world dataset on educational equity in New York City that includes data from 1,272 schools across 32 districts.

### 4.1 Loading MLOB package

First, install and load the MLOB package, which is available on CRAN:

**Figure:**
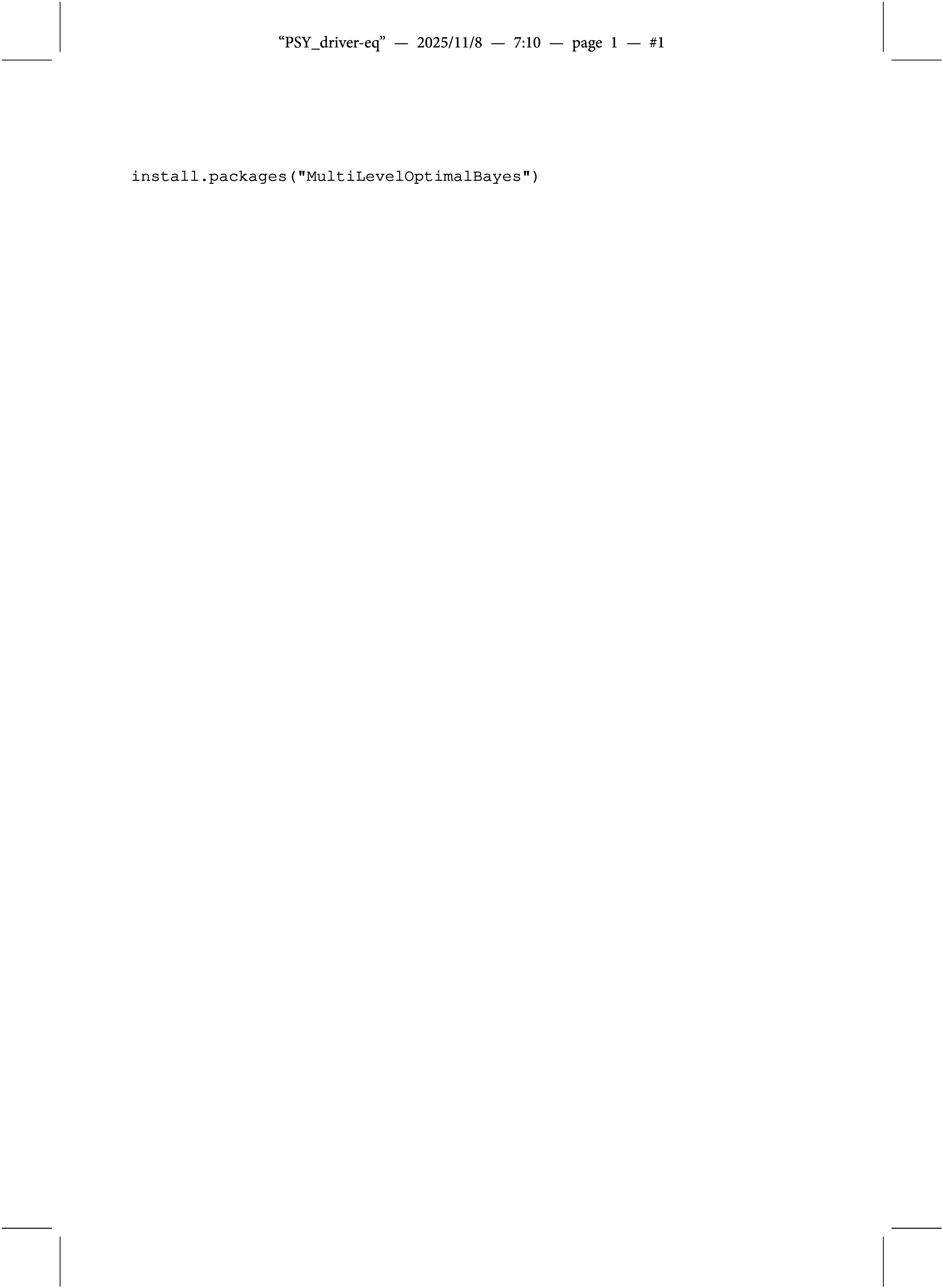

Alternatively, the development version can be installed from GitHub:

**Figure:**
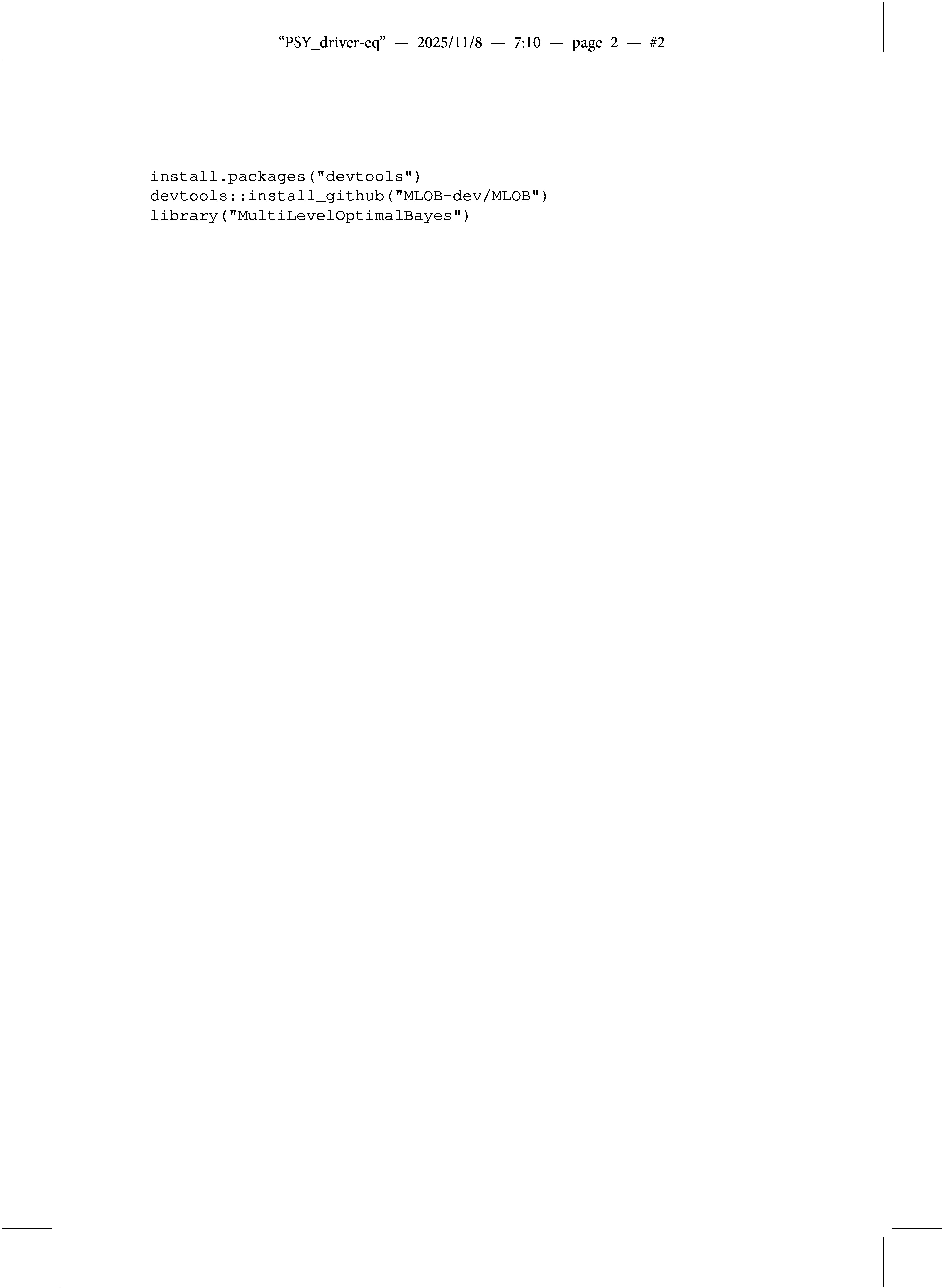

### 4.2 Loading and preparing the dataset

As mentioned earlier, we demonstrate how to use the MLOB package based on the PASSNYC dataset. The PASSNYC dataset is available on Kaggle.^3^ In the next step, load, clean, and convert the relevant variables of the PASSNYC dataset to numeric values:

**Figure:**
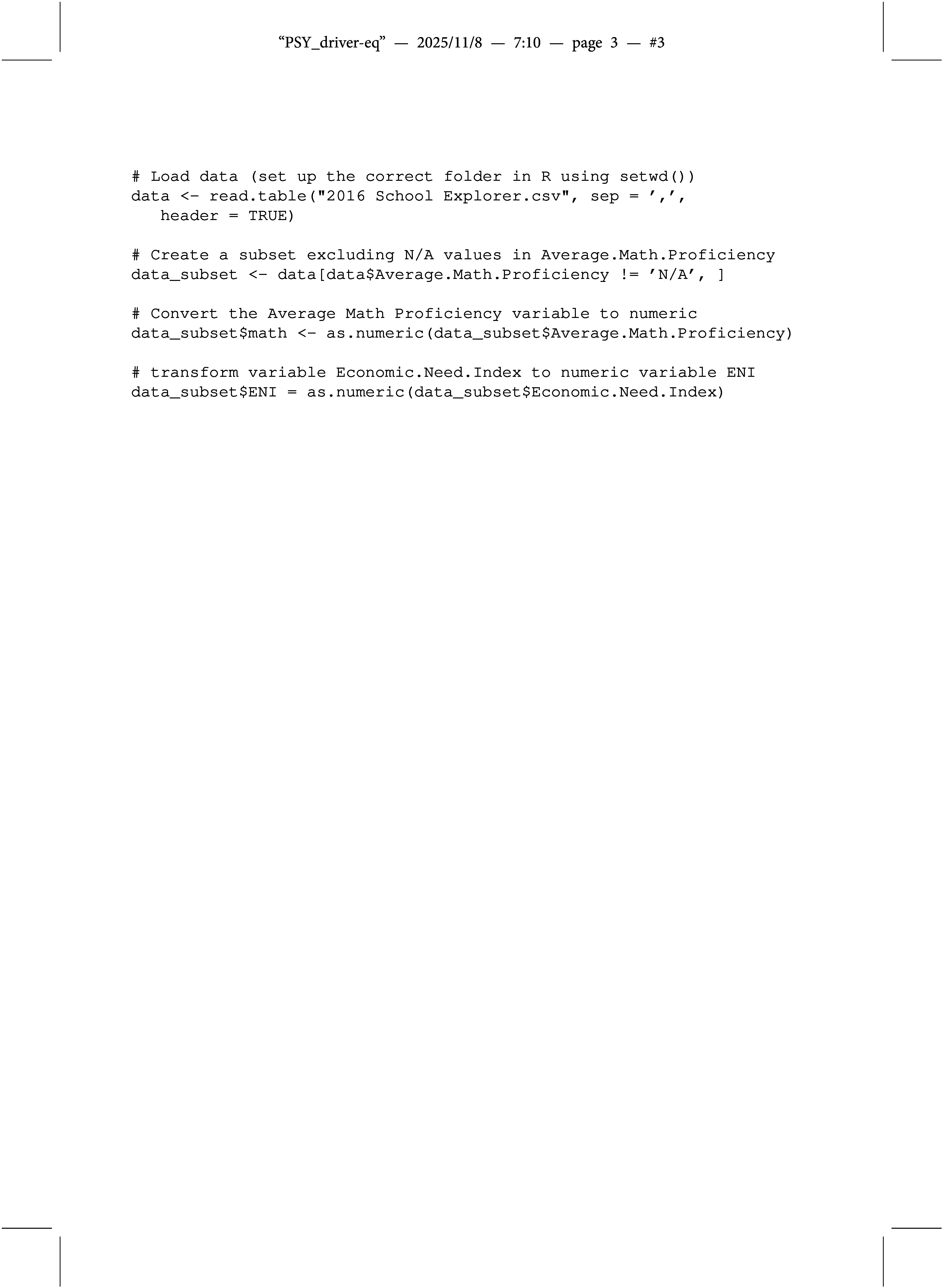

### 4.3 Estimating the between-group effect

We seek to obtain the contextual effect of economic need on average math proficiency using the regularized Bayesian estimator. For user convenience, the mlob() function follows a similar notation and works as simply as the linear regression function lm() in R. We specify District as the grouping variable. To ensure reproducibility, we set a random seed before processing the dataset. Since the dataset is unbalanced (i.e., the number of individuals per group varies), our procedure balances the data by randomly removing entities from larger groups to achieve equal group sizes. Setting a seed ensures that the same entities are removed each time the procedure is run, making the results fully replicable.

**Figure:**
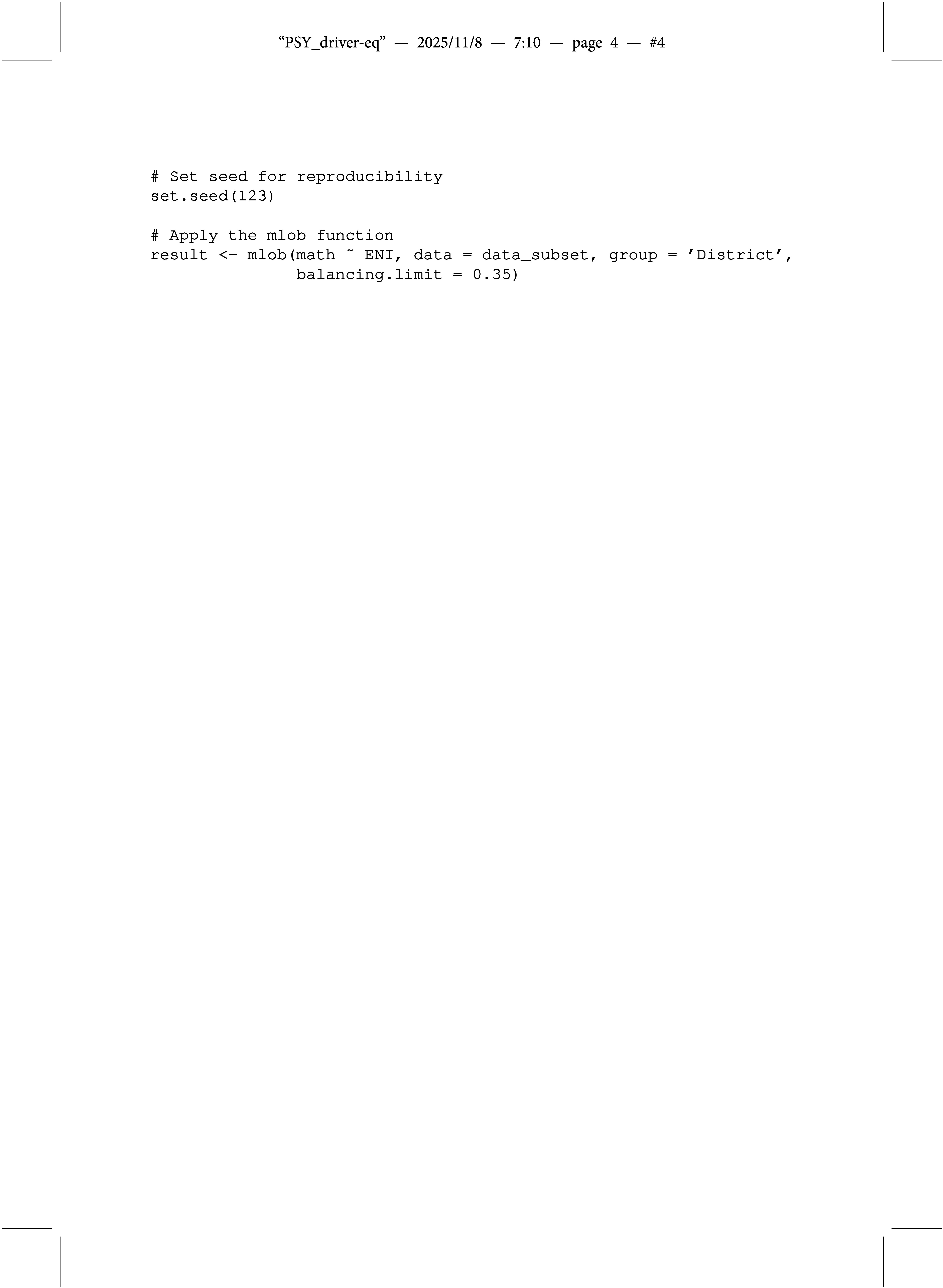

Warnings may indicate that the data are unbalanced and that a balancing procedure has been applied. The function also alerts the user if estimates may be unreliable due to a highly unbalanced structure. By default, if more than 20% of the data would need to be deleted to achieve balance (threshold adjustable via the 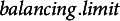 parameter), the function stops and issues a warning. While this procedure preserves the estimator’s assumptions, removing many observations or groups may affect the generalizability of the results.

### 4.4 Summary of results

The output of the customized summary() function follows the format of the summary(lm()) function and provides the estimated between-group effect ( 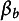 ) obtained with the regularized Bayesian estimator. For comparison, the summary() function also includes ML estimation results:

**Figure:**
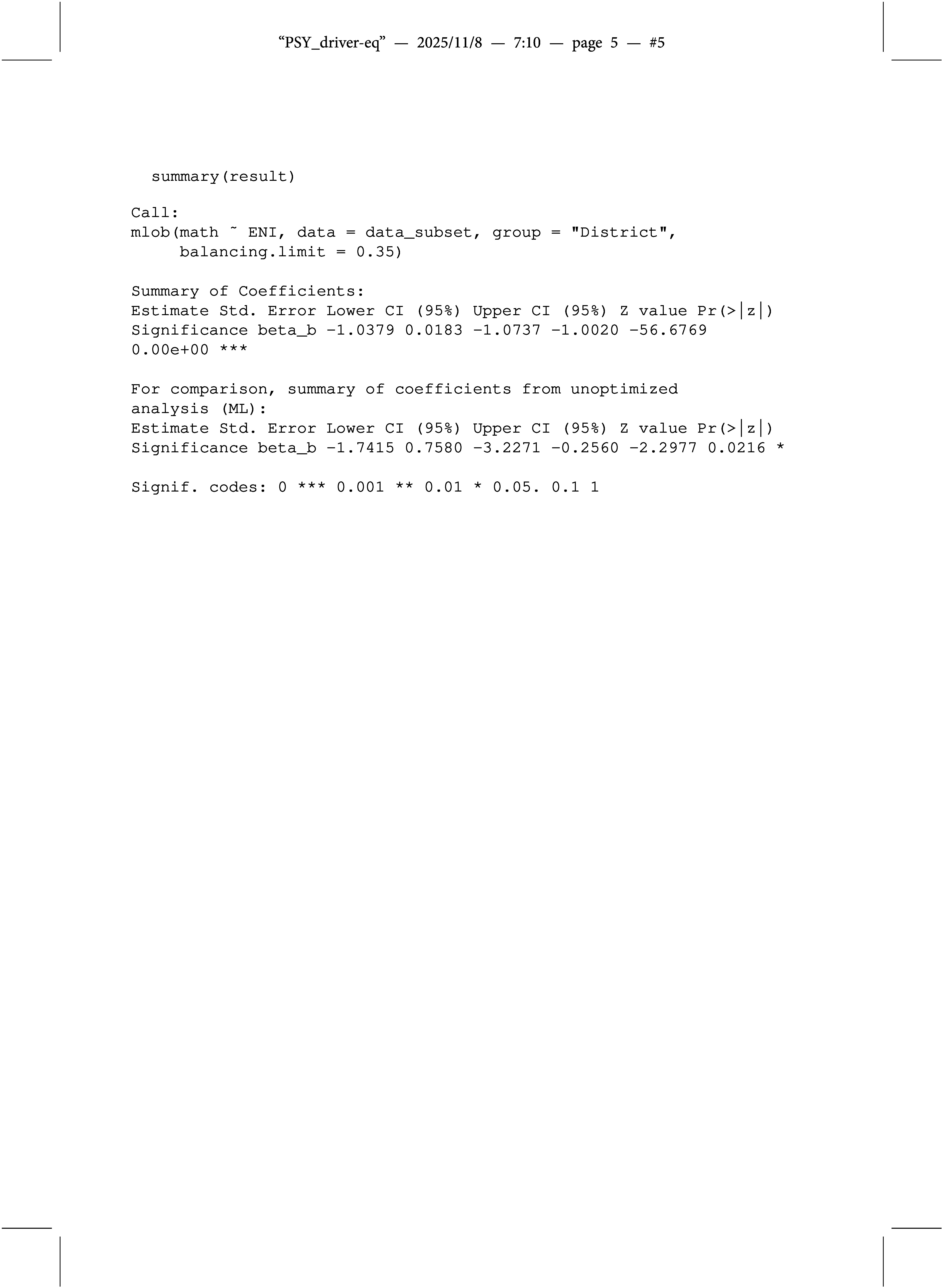

### 4.5 Interpretation

The results indicate that the regularized Bayesian estimator provides an estimate with a significantly lower standard error compared to the ML estimator. Notably, the between-group coefficient estimated by the regularized Bayesian estimator ( 
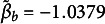
 ) is smaller in absolute terms than the one estimated by ML ( 
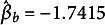
 ). The reduction in absolute magnitude suggests that ML may overestimate the effect due to its higher variance, whereas the regularized Bayesian estimator produces more reliable estimates, particularly in small samples. The between-group effect in this context represents how economic need, averaged at the district level, influences math proficiency across the districts of New York City. The negative coefficient suggests that districts with higher economic need tend to have lower average math proficiency. Given that the PASSNYC dataset is relatively small, containing 1,272 schools across 32 districts, the primary small-sample issue arises from the limited number of districts rather than the total number of schools. Since hierarchical models rely on the number of groups to estimate between-group effects, a small number of districts leads to increased variance in the estimated between-group coefficient. In this setting, the lower variance of the Bayesian estimator is particularly beneficial, as it enhances the reliability of the estimates. This highlights the advantages of the regularized Bayesian estimator in two-level latent variable models, especially with small datasets such as PASSNYC.

To draw a parallel with the previous section, we refer to Table 1, which summarizes the average RMSE and relative bias across different *n* and *J* and illustrates when regularized Bayesian or ML estimation is the preferable choice. A green color code is used to indicate the superior estimator for each scenario. Notably, in all analyzed cases, the newly developed estimator outperformed ML in terms of RMSE, further demonstrating its reliability in multilevel latent variable modeling. Therefore, even when the sample is sufficiently large, we recommend using our MLOB package, which offers both ML and regularized Bayesian estimations, allowing users to select the most appropriate method for their data. It is also important to consider degenerate cases where either the between-group or within-group effect is zero. In such cases, the mlob() function recommends using simpler models, such as ordinary least squares (OLS) or ML.

## 5 Discussion and conclusion

In this article, we thoroughly described and analyzed a regularized Bayesian estimator for multilevel latent variable models, which we optimized with respect to MSE performance, using the multilevel latent covariate model as an example. In addition, we derived an analytical expression for the standard error.

However, given our specific focus on small sample size, rather than using this standard error, it might be more reasonable to employ a resampling technique for accurately determining the standard error. As mentioned, one such effective method is a delete-d jackknife procedure. The main achievement lies in deriving an optimally regularized Bayesian estimator by seamlessly integrating the minimization of MSE with respect to the parameters of the prior distribution. Through graphical representations of the results, we highlighted the pronounced improvements that our approach garners over ML estimation, particularly in small samples.

The following contributions to the theoretical landscape are noteworthy. Primarily, we derived a distribution of the Bayesian estimator, enabling us to achieve further optimization of the MSE with respect to the parameters of the prior distribution for this estimator. Moreover, we proposed an algorithm to construct our optimally regularized Bayesian estimator. These theoretical achievements are mirrored by the results from our simulation study as detailed in the previous section. In a nutshell, from these results, significant performance improvements emerged for the optimally regularized Bayesian estimator compared to the ML estimator, particularly in situations characterized by small sample sizes and low ICCs. These advantages can be attributed to the way the estimator is constructed, which allows for some bias while actively minimizing the MSE.

Although our work focuses on Bayesian estimation, the utilization of prior information to enhance estimation is not exclusive to Bayesian methods. Similar means are taken by frequentist approaches. For example, the Bayesian estimator’s weighting parameter 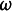 in Equation (8) achieves an effect analogous to the penalty in regularized structural equation modeling, as seen in Jacobucci et al. (2016). Similarly, the weighting parameter in the denominator of Equation (7) aligns with the concept of regularized consistent partial least squares estimation (e.g., Jung & Park, 2018).

While our research offers significant contributions, we also acknowledge limitations. The advantages of our method over ML estimation become less pronounced with larger sample sizes, indicating that our approach may be most beneficial in contexts with smaller samples. Another limitation of our approach lies in the locality of the search for the optimal MSE. Our optimization strategy within a 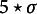 region ensures that the minimum MSE falls within this region with almost 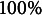 probability, although this is not guaranteed. Additionally, since the true MSE remains unknown, we rely on the estimated MSE, which provides a reliable approximation within the defined bound. However, the extrema of the real and estimated MSE do not always coincide. As a result, misspecification of the regularized Bayesian estimation is possible but extremely unlikely. Moreover, by reducing the 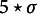 search region, we can control bias and select an optimal estimator within the reduced region. While this decreases the probability of finding the globally optimal MSE, it ensures that the estimator has a relative bias within a predefined threshold. In the degenerate case where the search region is zero, we obtain an exact ML estimator. This is a potential area for future research.

One more limitation is the assumption of equal group sizes, which simplifies the statistical problem. However, in practice, group sizes often vary (e.g., the number of students in classes). While our current approach does not directly account for unequal group sizes, one possible solution would be to average the group sizes and apply our estimator. It is important to note that our regularized Bayesian estimator formulas extend to non-integer values of *n*, allowing for this flexibility. This is also a potential area for future research. Nevertheless, our MLOB R package includes a built-in data-balancing mechanism that provides a practical solution for handling unequal group sizes. Notably, if more than 20% of the data would need to be deleted to achieve balance, the function stops and alerts the user.

Beyond these limitations, the regularized Bayesian estimator can be extended to three- and higher-level models. While our estimator has not yet been fully developed for such multilevel structures, these models could be implemented through an iterative application of the two-level estimator. One approach is to iteratively apply the regularized Bayesian estimator by reducing the model to two levels at a time, computing estimates, and then proceeding to the next pair of levels.

An extension for future simulation work is to explore a broader range of between-group parameter values, including near-zero 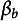 settings, to more fully assess performance under weak between-group effects. Future designs could also relax the constraints on ICC 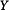 to investigate the estimator’s behavior in such scenarios.

Another possible extension is incorporating time as a predictor, enabling a longitudinal modeling framework for analyzing time-related trends. For example, the application of our regularized Bayesian estimator to the longitudinal dataset ChickWeight is included as a standard example in the MLOB R package. Such extensions provide promising directions for future research and further refinement of the regularized Bayesian estimator.

To conclude, our optimized Bayesian estimator, which sophistically balances bias reduction and variance minimization, offers improved precision in parameter estimation, particularly in small samples. Thus, our findings hold promising implications for multilevel latent variable modeling, and the demonstrated accuracy improvements due to optimized regularization underscore the practical value of our estimator. We aspired to empower researchers in psychology and related fields to utilize the benefits of our proposed estimator and use the newly developed mlob package in R, as demonstrated in the Section Step-by-Step Tutorial when dealing with small samples in fitting multilevel latent variable models.

By highlighting the efficacy of Bayesian strategies, we hope to inspire a paradigm shift in estimation techniques for small-sample scenarios. This shift could lead to more robust and informed modeling practices in the research community.

## Footnotes

1 In this case, optimized stands for  that minimizes the total error of an approximated denominator of the Bayesian estimator in Equation (7).

2 The method with the smallest MSE also has the smallest RMSE, and the reverse is also true.

3
https://www.kaggle.com/datasets/passnyc/data-science-for-good/data

## Appendix A

To derive a Bayesian estimator following Zitzmann, Helm, and Hecht (2021) indirect strategy, we start by adopting a gamma prior distribution for the inverse of the group-level variance of the predictor variable  :

(A.1)

where *a* and *b* are the parameters of the Gamma distribution. For better interpretability, we employ a reparameterization of  and  leading to

(A.2)

Similarly, the likelihood for the inverse of the group-level variance is

(A.3)

with  being an estimate of the group-level variance. To get an inverse-gamma posterior, we combine Equations (A.2) and (A.3) and yield

(A.4)

As demonstrated by Zitzmann, Lüdtke, et al. (2021) in Appendix C, an approximation for the mean of this distribution can be derived as follows:

(A.5)

With Equation (A.5), the Bayesian expected a posteriori (EAP) estimate is defined. We specify the weighting parameter  from Equation (A.5) as

(A.6)

This formula minimizes the total error of the approximation of  from Equation (A.5), making it optimal.

Note that  is defined in Equation (A.6) as a function of sample size, or more precisely, as a function of the number of groups *J*.

Asymptotically, when  ,  converges to 1. Thus,  becomes equal to  in this case.

To derive the new estimator, we take Equation (6) and replace  , with its Bayesian EAP as defined in Equation (A.5). This gives

(A.7)

## Appendix B

To compute an estimate of the group-level covariances, we apply the formulas from Zitzmann, Lüdtke, et al. (2021), starting from the decompositions:

(B.1)

(B.2)

We assume that  and  are uncorrelated and both independently identically normally distributed. The same assumptions are considered for *Y*.

Next, we define (manifest) group means for both *X* and *Y* as

(B.3)

(B.4)

Then, the overall means are

(B.5)

(B.6)

The sums of squared deviations of the group means from the overall mean (SSA) and of the individual values from the group means (SSD) for *X* are

(B.7)

(B.8)

The same equations hold for *Y*. And the cross products of *Y* and *X* are

(B.9)

(B.10)

Zitzmann, Lüdtke, et al. (2021) derived the relations between the sum of squared deviations of *X* and the within- and between-group variances as

(B.11)

(B.12)

Combining Equations (B.11) and (B.12) with Equations (B.7) and (B.8), we yield an estimate of the group-level variance of *X*:

(B.13)

Note that this estimator may not be optimal, because estimates may not be positive. To address this issue, Chung et al. (2013) introduced a maximum penalized likelihood (MPL) approach for estimating this parameter. This method mitigates the problem of boundary estimates, specifically preventing the occurrence of negative estimated group-level variances. In our approach, we used the estimator from Equation (B.13), due to the transformation in the further steps and no anomalies were found during the extensive simulations.

Zitzmann, Lüdtke, et al. (2021) also derived how the sum of squared deviations of cross products of *X* and *Y* can be expressed in terms of their within- and between-group covariances:

(B.14)

(B.15)

This means that the estimator for the group-level covariance  can be obtained from Equations (B.9), (B.10), (B.14), and (B.15) as

(B.16)

So far, we have derived both the numerator and the denominator of the ML estimator and, partly, of the Bayesian estimator in Equation (7). But how can we use these derivations? Our aim is to minimize the MSE of the Bayesian estimator, and to do this, we need to know the mean and the variance of the estimator. One way to find them is to compute the estimator’s distribution.

We begin with the derivation of the distributions of group-level variance of *X* and the group-level covariance between *X* and *Y*. To this end, two new variables are defined. The  merges all the elements of predictor sample together with its means into one vector of length  , and  combines all the elements of the dependent variable and its means:

(B.17)

(B.18)

Using these newly defined variables, we can rewrite the estimators for the group-level variance and the covariance  in matrix form:

(B.19)

(B.20)

With the same coefficient matrix *A* for both defined in Equation (F.1) of Appendix F. Note that matrix *A* is diagonal.

Thus,  and  are quadratic forms of the sample elements and their means. If the equations consist only of second-order terms of normally distributed random variables, we can interpret  and  as the weighted sums of  , and thus gamma-distributed random variables. However, the distribution of such a quadratic form is highly complicated in the general case. Therefore, we apply a transformation to yield weighted sum of squares (without interaction terms) of iid normal random variables.

Firstly, we compute the distribution of  and  , using the previously made assumptions about *X* and *Y*:

(B.21)

(B.22)

Where  is a vector of ones of size  . Also, note the following important facts:

- each element of  and  has the same mean;
- the sum of coefficients defined by matrix *A* in Equation (F.1) of Appendix F is zero.

As a result, when we demean Equations (B.19) and (B.20), these means sum up to zero. To demonstrate it, define  and  and all their elements as the demeaned counterparts of  and  , respectively:

(B.23)

Show that  and  are both zeros:

(B.24)

(B.25)

Plug the expressions from Equation (B.23) into Equations (B.19) and (B.20), and remind that the sum of coefficients of matrix *A* is zero:

(B.26)

(B.27)

Hence, it is irrelevant for  and  whether  and  have non-zero means or not, they always cancel out. So, we do not lose generality by assuming  and  .

and  are defined in Equations (F.3) and (F.4) of Appendix F. These matrices are symmetric and positive semi-definite as covariance matrices. Therefore, their square roots will have only real entries (Horn & Johnson, 2013). Using the matrices, we can transform  to

(B.28)

Where  follows the standard (multivariate) normal distribution, which has the identity matrix  as the covariance matrix. Following the rationale that led to Equation (B.28), we define a square root of the covariance matrix  by using its spectral decomposition as

(B.29)

Where  is a matrix of eigenvectors and it is orthogonal (  ), because  is a real symmetric matrix by its nature (Horn & Johnson, 2013). Matrix  is a diagonal matrix of eigenvalues. These eigenvalues are non-negative, because  is positive-semidefinite (Horn & Johnson, 2013). Thus, we may denote the square root of  as  , which is just a diagonal matrix with real square roots of each element of  . This helps us to define the matrix  :

(B.30)

Indeed, we have

(B.31)

The eigenvalues of  are as follows:

- ,  eigenvalues;
- ,  eigenvalues;
- ,  eigenvalues;
- , 1 eigenvalue.

 , a diagonal matrix, is composed of the eigenvalues in this order. Matrix  is presented in Equation (F.2) of Appendix F. Due to its bulkiness, we provide  for the case  and  , but it could be expanded upon demand.

We can now plug the decomposition of  into Equation (B.28) so that it becomes

(B.32)

(B.33)

where  . Thus, the orthogonality of matrix  kept the standard normal distribution of the new variable  . Since the internal right-hand side of Equation (B.33),  , is diagonal, we indeed managed to represent  as a weighted sum of squares of independent normally distributed random variables, that is, a weighted sum of  -distributed random variables.

## Appendix C

Similarly to the transformation of the group-level variance of *X*, which was introduced in Appendix B, we continue with the description of the transformation of the group-level covariance of *X* and *Y* as this is partially similar. We start from Equation (B.20) in Appendix B and use the previously defined covariance matrices  and  (Equations (B.21) and (B.22) in Appendix B):

(C.1)

where  is a new random vector that follows the multivariate standard normal distribution. For further transformation, we also introduce the spectral decomposition of covariance matrix  and its square root as

(C.2)

(C.3)

where  is a matrix of eigenvectors of  . It turns out to be equal to  , therefore sharing its property of orthogonality. We will further refer to them as  (see Equation (F.2) in Appendix F).

Matrix  consists of (non-negative) eigenvalues of  on the diagonal (because of the positive-semidefiniteness of  ). Its square root matrix,  , is also diagonal, with non-negative square roots of eigenvalues on the main diagonal. We can compute the eigenvalues of  in closed form and thus define matrix  by

- ,  eigenvalues;
- ,  eigenvalues;
- ,  eigenvalues;
- , 1 eigenvalue.

For the next step, we plug in the decompositions Equation (B.30) of Appendix B and Equation (C.3) into Equation (C.1) and obtain

(C.4)

(C.5)

where  . Thus, the distribution of the new variable  is standard normal because of the orthogonality of the matrix *V*. Additionally, the inner right-hand side of Equation (C.5),  , is diagonal due to its construction. Comparing Equations (B.33) and (C.5), one might be inclined to see the distinct similarities and the claim to also represent  as a weighted sum of squares of independent normally distributed random variables. However, this is not true.  and  are different random vectors, and thus, we continue the transformation by defining a new aggregated variable:

(C.6)

with the distribution of *H* being  . Its covariance matrix  is defined as follows:

(C.7)

We already showed that  and  as well. Before calculation of  , we additionally define  in Equation (F.5) of Appendix F in a manner similar to Equations (F.3) and (F.4). Then, the spectral decomposition of  becomes

(C.8)

(C.9)

where matrix *V* is the same as in decompositions of  in Equation (B.29) from Appendix B and  in Equation (C.2). Matrix  is diagonal with non-negative eigenvalues of positive-semidefinite matrix  (Horn & Johnson, 2013). Thus, the square root matrix,  , is diagonal with non-negative square roots of eigenvalues on the main diagonal. The eigenvalues of  that define matrix  are in the closed form:

- ,  eigenvalues;
- ,  eigenvalues;
- ,  eigenvalues;
- , 1 eigenvalue.

Next, we use the generalized inverses of matrices  and  , as described by Penrose (1955), since they include zero eigenvalues and are not invertible. These matrices are denoted as  and  and include the inverse of diagonal elements that are invertible and zeros otherwise.

Using all this, the covariance  is computed as

(C.10)

This result is used to fully define the covariance matrix of *H*:

(C.11)

and its spectral decomposition:

(C.12)

where the closed-form solutions for both the matrix of eigenvalues  and the orthogonal matrix of eigenvectors  .  is

(C.13)

Matrix  is defined in Equation (F.6) of Appendix F. Both matrices follow the same properties as their predecessor:  is diagonal with non-negative eigenvalues, and  is orthogonal.

After exposing the new composite vector *H* and its covariance matrix  , we can rewrite Equation (C.5) as

(C.14)

with coefficient matrix *Q* defined as

(C.15)

Note that *Q* is designed to keep the symmetry of Equation (C.14). Including the square root of the covariance matrix leads to

(C.16)

where  is a vector of independent normally distributed variables. Using the decomposition of  from Equation (C.12), denoting a square root of  as  , and plugging both terms into Equation (C.16) yields:

(C.17)

(C.18)

with  —a multivariate standard normally distributed random vector, as  is orthogonal. Furthermore, since matrix  is diagonal, the estimator of the group-level covariance  is now represented as a weighted sum of squares of independent normally distributed random variables, that is, a weighted sum of  -distributed random variables. Thus, at this point, we achieved our aim of transforming  .

## Appendix D

Here, we derive the distributions of the ML and the Bayesian estimator. To this end, we start by calculating the distributions of sample group-level covariances  and  in Equations (10) and (11), respectively. According to Welch (1947) and Satterthwaite (1946), we can approximate these sums as a generic Gamma distribution with parameters:

(D.1)

(D.2)

Notice that each element in the sums  and  is scaled. The scales are defined by diagonal matrices  (for  ) and  (for  ). Let us denote their diagonal elements as  and  , respectively. Then, we can express the distributions of  and  as

(D.3)

(D.4)

Using these distributions, we can find the distribution of the ML estimator. It is well known that the ratio of two independent gamma-distributed random variables follows *F* distribution. The independence of  and  is not directly clear, but it follows from the approximation of the sum of Gamma-distributions. Therefore, the ML estimator’s distribution is

(D.5)

Next, we derive the distribution of the Bayesian estimator. Since it includes the two parameters  and  , we need to adjust the process of derivation and find the distribution of the denominator first.

The denominator is  and consists of a stochastic part  and deterministic part  . To sum them up, we replace the deterministic part with the sequence of random variables  , which converges (in probability) to this deterministic part:

(D.6)

Further, we substitute  with  and yield a sum of gamma-distributed random variables. Using once more the approach from Welch (1947) and Satterthwaite (1946), we compute a sum as a new sequence of random variables that follows a Gamma distribution with parameters  and  :

(D.7)

(D.8)

The limit is the Gamma  distribution with parameters:

(D.9)

(D.10)

Using the derived distribution of the denominator, similarly to the ML estimator, we yield the total distribution of the Bayesian estimator:

(D.11)

After computing the distributions of the ML estimator (Equation (D.5)) and the Bayesian estimator (Equation (D.11)), we use them to calculate biases and variances of the estimators and thus their MSEs as

(D.12)

(D.13)

## Appendix E: Estimation algorithm

Finally, we introduce a novel and practical algorithm based on the theoretical investigations made in the main part of the article. This algorithm aims to provide an efficient and effective solution for computing the regularized Bayesian estimator:

1. Input data: *n*, *J*,  , and
2. Define matrix *A* from Equation (F.1) of Appendix F.
3. Calculate the (manifest) group means:  of *X* from Equation (B.3) in Appendix B and  of *Y* from Equation (B.4) in Appendix B.
4. Calculate the overall means:  of *X* from Equation (B.5) in Appendix B and  of *Y* from Equation (B.6) in Appendix B.
5. Compute  from Equation (B.13) in Appendix B and  from Equation (B.16) in Appendix B as well as
  (E.1)
  (E.2)
  (E.3)
  (E.4)
6. Find the ML estimator  from Equation (6).
7. Compute diagonal matrices of eigenvalues  (page 44),  (page 46),  (page 48), and matrix of eigenvectors *V* from Equation (F.2) of Appendix F.
8. Calculate the square root matrices  and  .
9. Compute the diagonal matrix of coefficients
10. Calculate matrix *Q* from Equation (C.15) in Appendix C.
11. Compute the diagonal matrix of eigenvalues  from Equation (C.13) of Appendix C and eigenvectors matrix  from Equation (F.6) of Appendix F.
12. Calculate the square root matrix
13. Compute the diagonal matrix of coefficients
14. Compute the coefficients  ,  ,  and  (note that  is a vector of ones):
  (E.5)
  (E.6)
  (E.7)
  (E.8)
15. Define vectors *W* and  , with the values of  and  that specify grid search region. For example, *W* goes from  to  by steps of  , and  goes from  to  by steps of
16. Compute the MSE for each value of *W* and  , whereby  should be substituted with  . The final formula is delineated as
  (E.9)
17. Find the minimum MSE and indexes  and  that provide this minimum.
18. Define the optimal parameters  and
19. Compute the optimally regularized Bayesian estimator as
  (E.10)

## Appendix F: Matrices

(F.1)

(F.2)

(F.3)

(F.4)

(F.5)

(F.6)

## Appendix G: Tables

**Table 2:** RMSE values of the ML (RMSE  ) and the Bayesian estimators (RMSE  represents the Bayesian with  and RMSE  represents the Bayesian with  ) for ICC  and different values of *n*, *J*,  , and

				RMSE	RMSE	RMSE					RMSE	RMSE	RMSE
5	5	0.2	0.2	57.587	2.725	2.667	15	20	0.5	0.7	6.992	0.913	0.913
5	5	0.2	0.5	529.69	3.065	3.036	15	20	0.6	0.2	24.893	0.868	0.872
5	5	0.2	0.7	93.937	3.475	3.462	15	20	0.6	0.5	31.363	0.863	0.864
5	5	0.5	0.2	29.285	2.644	2.613	15	20	0.6	0.7	271.245	0.883	0.883
5	5	0.5	0.5	32.474	3.07	3.043	15	30	0.2	0.2	24.704	0.67	0.676
5	5	0.5	0.7	108.22	3.458	3.426	15	30	0.2	0.5	14.967	0.702	0.707
5	5	0.6	0.2	37.614	2.79	2.755	15	30	0.2	0.7	66.872	0.769	0.775
5	5	0.6	0.5	38.324	3.036	3.002	15	30	0.5	0.2	85.611	0.679	0.681
5	5	0.6	0.7	246.624	3.4	3.393	15	30	0.5	0.5	17.079	0.703	0.704
5	10	0.2	0.2	103.585	1.868	1.862	15	30	0.5	0.7	18.208	0.749	0.748
5	10	0.2	0.5	18.639	2.121	2.133	15	30	0.6	0.2	15.823	0.744	0.743
5	10	0.2	0.7	68.983	2.348	2.346	15	30	0.6	0.5	11.853	0.72	0.719
5	10	0.5	0.2	21.904	1.881	1.872	15	30	0.6	0.7	9.818	0.741	0.739
5	10	0.5	0.5	172.495	2.051	2.039	15	40	0.2	0.2	6.608	0.516	0.525
5	10	0.5	0.7	85.472	2.383	2.392	15	40	0.2	0.5	3.685	0.546	0.551
5	10	0.6	0.2	65.174	1.911	1.894	15	40	0.2	0.7	18.378	0.585	0.593
5	10	0.6	0.5	19.356	2.129	2.124	15	40	0.5	0.2	15.753	0.61	0.611
5	10	0.6	0.7	553.141	2.315	2.313	15	40	0.5	0.5	5.633	0.607	0.605
5	20	0.2	0.2	32.3	1.37	1.364	15	40	0.5	0.7	25.081	0.606	0.603
5	20	0.2	0.5	186.452	1.486	1.491	15	40	0.6	0.2	9.669	0.652	0.648
5	20	0.2	0.7	31.417	1.633	1.652	15	40	0.6	0.5	6.565	0.61	0.607
5	20	0.5	0.2	528.303	1.302	1.313	15	40	0.6	0.7	13.398	0.632	0.629
5	20	0.5	0.5	15.767	1.38	1.376	30	5	0.2	0.2	346.81	1.549	1.554
5	20	0.5	0.7	81.714	1.614	1.612	30	5	0.2	0.5	697.82	1.646	1.649
5	20	0.6	0.2	84.956	1.347	1.347	30	5	0.2	0.7	841.537	1.734	1.732
5	20	0.6	0.5	22.968	1.379	1.378	30	5	0.5	0.2	44.781	1.552	1.554
5	20	0.6	0.7	70.052	1.57	1.58	30	5	0.5	0.5	41.708	1.557	1.56
5	30	0.2	0.2	25.439	1.087	1.098	30	5	0.5	0.7	116.407	1.712	1.718
5	30	0.2	0.5	39.795	1.142	1.14	30	5	0.6	0.2	89.971	1.519	1.523
5	30	0.2	0.7	157.449	1.337	1.343	30	5	0.6	0.5	51.606	1.591	1.593
5	30	0.5	0.2	17.714	1.107	1.113	30	5	0.6	0.7	111.256	1.604	1.611
5	30	0.5	0.5	65.436	1.175	1.169	30	10	0.2	0.2	125.74	1.067	1.072
5	30	0.5	0.7	20.967	1.249	1.248	30	10	0.2	0.5	44.729	1.087	1.091
5	30	0.6	0.2	112.352	1.104	1.109	30	10	0.2	0.7	28.094	1.133	1.136
5	30	0.6	0.5	24.527	1.181	1.183	30	10	0.5	0.2	11.672	1.057	1.061
5	30	0.6	0.7	31.858	1.241	1.25	30	10	0.5	0.5	25.1	1.076	1.078
5	40	0.2	0.2	42.185	0.979	0.983	30	10	0.5	0.7	164.174	1.13	1.131
5	40	0.2	0.5	53.56	1.054	1.053	30	10	0.6	0.2	40.099	1.044	1.045
5	40	0.2	0.7	15.499	1.082	1.086	30	10	0.6	0.5	68.118	1.047	1.05
5	40	0.5	0.2	115.685	0.987	0.987	30	10	0.6	0.7	122.808	1.088	1.092
5	40	0.5	0.5	32.061	0.998	0.994	30	20	0.2	0.2	4.08	0.587	0.597
5	40	0.5	0.7	28.321	1.161	1.166	30	20	0.2	0.5	6.696	0.561	0.571
5	40	0.6	0.2	34.223	0.973	0.97	30	20	0.2	0.7	8.71	0.587	0.597
5	40	0.6	0.5	107.287	1.018	1.016	30	20	0.5	0.2	6.382	0.647	0.646
5	40	0.6	0.7	41.419	1.044	1.047	30	20	0.5	0.5	66.702	0.642	0.642
15	5	0.2	0.2	205.555	1.676	1.667	30	20	0.5	0.7	6.12	0.647	0.644
15	5	0.2	0.5	290.677	1.777	1.762	30	20	0.6	0.2	9.591	0.693	0.689
15	5	0.2	0.7	89.916	1.946	1.942	30	20	0.6	0.5	11.39	0.669	0.665
15	5	0.5	0.2	96.434	1.599	1.597	30	20	0.6	0.7	2.793	0.705	0.702
15	5	0.5	0.5	61.309	1.747	1.742	30	30	0.2	0.2	0.981	0.342	0.353
15	5	0.5	0.7	83.573	1.936	1.926	30	30	0.2	0.5	0.794	0.322	0.333
15	5	0.6	0.2	34.357	1.622	1.61	30	30	0.2	0.7	2.281	0.331	0.341
15	5	0.6	0.5	111.232	1.742	1.739	30	30	0.5	0.2	0.973	0.503	0.499
15	5	0.6	0.7	328.599	1.904	1.903	30	30	0.5	0.5	2.255	0.476	0.472
15	10	0.2	0.2	216.574	1.186	1.184	30	30	0.5	0.7	1.147	0.494	0.491
15	10	0.2	0.5	2961.914	1.25	1.249	30	30	0.6	0.2	0.815	0.558	0.55
15	10	0.2	0.7	93.279	1.271	1.278	30	30	0.6	0.5	0.745	0.541	0.533
15	10	0.5	0.2	30.459	1.195	1.195	30	30	0.6	0.7	1.736	0.564	0.558
15	10	0.5	0.5	120.55	1.208	1.209	30	40	0.2	0.2	0.621	0.231	0.24
15	10	0.5	0.7	19.802	1.288	1.291	30	40	0.2	0.5	3.259	0.241	0.252
15	10	0.6	0.2	135.805	1.178	1.181	30	40	0.2	0.7	0.651	0.261	0.272
15	10	0.6	0.5	40.038	1.221	1.226	30	40	0.5	0.2	0.708	0.443	0.435
15	10	0.6	0.7	35.817	1.265	1.268	30	40	0.5	0.5	0.572	0.441	0.435
15	20	0.2	0.2	24.678	0.878	0.883	30	40	0.5	0.7	1.291	0.432	0.427
15	20	0.2	0.5	166.166	0.875	0.878	30	40	0.6	0.2	1.296	0.522	0.514
15	20	0.2	0.7	19.525	0.936	0.939	30	40	0.6	0.5	0.731	0.509	0.502
15	20	0.5	0.2	43.648	0.899	0.903	30	40	0.6	0.7	0.475	0.508	0.501
15	20	0.5	0.5	62.277	0.879	0.878

**Table 3:** RMSE values of the ML (RMSE  ) and the Bayesian estimators (RMSE  represents the Bayesian with  and RMSE  represents the Bayesian with  ) for ICC  and different values of *n*, *J*,  , and

				RMSE	RMSE	RMSE					RMSE	RMSE	RMSE
5	5	0.2	0.2	33.935	2.436	2.383	15	20	0.5	0.7	2.45	0.511	0.51
5	5	0.2	0.5	612.83	2.858	2.853	15	20	0.6	0.2	24.405	0.578	0.578
5	5	0.2	0.7	258.045	3.069	3.057	15	20	0.6	0.5	1.927	0.551	0.548
5	5	0.5	0.2	46.967	2.389	2.341	15	20	0.6	0.7	3.717	0.547	0.544
5	5	0.5	0.5	61.524	2.63	2.607	15	30	0.2	0.2	1.268	0.257	0.271
5	5	0.5	0.7	41.284	2.988	2.976	15	30	0.2	0.5	0.733	0.265	0.28
5	5	0.6	0.2	38.72	2.449	2.383	15	30	0.2	0.7	0.807	0.308	0.321
5	5	0.6	0.5	346.286	2.657	2.625	15	30	0.5	0.2	0.723	0.42	0.416
5	5	0.6	0.7	58.937	3.06	3.049	15	30	0.5	0.5	3.031	0.417	0.413
5	10	0.2	0.2	176.892	1.591	1.571	15	30	0.5	0.7	1.083	0.421	0.418
5	10	0.2	0.5	20.44	1.737	1.736	15	30	0.6	0.2	0.657	0.478	0.472
5	10	0.2	0.7	49.498	1.994	1.99	15	30	0.6	0.5	1.69	0.475	0.47
5	10	0.5	0.2	55.096	1.52	1.509	15	30	0.6	0.7	0.588	0.47	0.464
5	10	0.5	0.5	230.062	1.618	1.613	15	40	0.2	0.2	0.577	0.19	0.202
5	10	0.5	0.7	62.571	1.865	1.86	15	40	0.2	0.5	1.869	0.207	0.22
5	10	0.6	0.2	17.002	1.57	1.565	15	40	0.2	0.7	15.892	0.229	0.24
5	10	0.6	0.5	20.908	1.661	1.663	15	40	0.5	0.2	1.213	0.381	0.376
5	10	0.6	0.7	180.241	1.756	1.742	15	40	0.5	0.5	0.391	0.383	0.378
5	20	0.2	0.2	728.749	1.06	1.063	15	40	0.5	0.7	0.373	0.382	0.378
5	20	0.2	0.5	105.743	1.088	1.085	15	40	0.6	0.2	0.396	0.439	0.433
5	20	0.2	0.7	108.22	1.278	1.273	15	40	0.6	0.5	0.339	0.44	0.435
5	20	0.5	0.2	26.338	1.017	1.01	15	40	0.6	0.7	0.34	0.441	0.437
5	20	0.5	0.5	11.918	1.018	1.022	30	5	0.2	0.2	25.285	1.216	1.216
5	20	0.5	0.7	58.23	1.206	1.208	30	5	0.2	0.5	38.692	1.278	1.286
5	20	0.6	0.2	1378.614	1.001	1.005	30	5	0.2	0.7	135.292	1.247	1.248
5	20	0.6	0.5	39.003	1.061	1.057	30	5	0.5	0.2	20.224	1.135	1.136
5	20	0.6	0.7	123.476	1.104	1.11	30	5	0.5	0.5	40.565	1.21	1.212
5	30	0.2	0.2	52.669	0.793	0.801	30	5	0.5	0.7	46.002	1.159	1.163
5	30	0.2	0.5	20.106	0.836	0.832	30	5	0.6	0.2	124.16	1.127	1.129
5	30	0.2	0.7	14.304	0.926	0.928	30	5	0.6	0.5	12.079	1.129	1.13
5	30	0.5	0.2	11.626	0.769	0.763	30	5	0.6	0.7	46.667	1.198	1.201
5	30	0.5	0.5	18.425	0.789	0.786	30	10	0.2	0.2	4.575	0.61	0.621
5	30	0.5	0.7	33.711	0.792	0.8	30	10	0.2	0.5	4.977	0.628	0.64
5	30	0.6	0.2	14.777	0.789	0.793	30	10	0.2	0.7	29.187	0.651	0.664
5	30	0.6	0.5	14.068	0.82	0.819	30	10	0.5	0.2	2.935	0.629	0.63
5	30	0.6	0.7	50.97	0.858	0.854	30	10	0.5	0.5	12.047	0.665	0.665
5	40	0.2	0.2	13.05	0.616	0.625	30	10	0.5	0.7	6.835	0.68	0.681
5	40	0.2	0.5	6.66	0.655	0.661	30	10	0.6	0.2	3.711	0.684	0.684
5	40	0.2	0.7	322.906	0.757	0.759	30	10	0.6	0.5	10.482	0.679	0.676
5	40	0.5	0.2	12.974	0.642	0.642	30	10	0.6	0.7	6.904	0.667	0.667
5	40	0.5	0.5	12.791	0.662	0.655	30	20	0.2	0.2	0.505	0.227	0.243
5	40	0.5	0.7	8.006	0.711	0.712	30	20	0.2	0.5	0.479	0.223	0.242
5	40	0.6	0.2	35.647	0.693	0.699	30	20	0.2	0.7	0.592	0.235	0.252
5	40	0.6	0.5	13.025	0.661	0.66	30	20	0.5	0.2	0.441	0.395	0.391
5	40	0.6	0.7	25.894	0.703	0.701	30	20	0.5	0.5	0.6	0.4	0.395
15	5	0.2	0.2	32.744	1.411	1.402	30	20	0.5	0.7	0.437	0.394	0.39
15	5	0.2	0.5	823.55	1.494	1.497	30	20	0.6	0.2	18.717	0.458	0.452
15	5	0.2	0.7	13462.32	1.654	1.651	30	20	0.6	0.5	0.577	0.466	0.46
15	5	0.5	0.2	100.543	1.402	1.394	30	20	0.6	0.7	0.451	0.462	0.456
15	5	0.5	0.5	12.623	1.392	1.388	30	30	0.2	0.2	0.344	0.162	0.174
15	5	0.5	0.7	238.948	1.459	1.458	30	30	0.2	0.5	0.345	0.163	0.176
15	5	0.6	0.2	169.018	1.356	1.359	30	30	0.2	0.7	0.347	0.168	0.181
15	5	0.6	0.5	97.213	1.343	1.343	30	30	0.5	0.2	0.341	0.369	0.363
15	5	0.6	0.7	25.553	1.525	1.519	30	30	0.5	0.5	0.511	0.375	0.37
15	10	0.2	0.2	30.52	0.852	0.855	30	30	0.5	0.7	0.326	0.372	0.368
15	10	0.2	0.5	37.813	0.877	0.884	30	30	0.6	0.2	0.332	0.43	0.424
15	10	0.2	0.7	17.617	0.9	0.901	30	30	0.6	0.5	0.319	0.433	0.428
15	10	0.5	0.2	8.591	0.842	0.846	30	30	0.6	0.7	0.308	0.433	0.429
15	10	0.5	0.5	28.307	0.863	0.866	30	40	0.2	0.2	0.292	0.16	0.167
15	10	0.5	0.7	16.876	0.838	0.84	30	40	0.2	0.5	0.292	0.159	0.165
15	10	0.6	0.2	12.698	0.84	0.842	30	40	0.2	0.7	0.293	0.16	0.168
15	10	0.6	0.5	18.314	0.833	0.833	30	40	0.5	0.2	0.279	0.359	0.354
15	10	0.6	0.7	17.259	0.834	0.835	30	40	0.5	0.5	0.272	0.36	0.356
15	20	0.2	0.2	4.809	0.437	0.449	30	40	0.5	0.7	0.269	0.362	0.358
15	20	0.2	0.5	14.818	0.448	0.459	30	40	0.6	0.2	0.266	0.421	0.418
15	20	0.2	0.7	5.329	0.486	0.498	30	40	0.6	0.5	0.268	0.421	0.417
15	20	0.5	0.2	1.404	0.525	0.524	30	40	0.6	0.7	0.261	0.423	0.42
15	20	0.5	0.5	1.637	0.518	0.517

**Table 4:** RMSE values of the ML (RMSE  ) and the Bayesian estimators (RMSE  represents the Bayesian with  and RMSE  represents the Bayesian with  ) for ICC  and different values of *n*, *J*,  , and

				RMSE	RMSE	RMSE					RMSE	RMSE	RMSE
5	5	0.2	0.2	42.506	1.716	1.658	15	20	0.5	0.7	0.202	0.255	0.261
5	5	0.2	0.5	18.529	1.853	1.828	15	20	0.6	0.2	0.196	0.282	0.287
5	5	0.2	0.7	19.436	1.959	1.943	15	20	0.6	0.5	0.188	0.283	0.288
5	5	0.5	0.2	17.082	1.664	1.634	15	20	0.6	0.7	0.177	0.284	0.291
5	5	0.5	0.5	150.933	1.746	1.725	15	30	0.2	0.2	0.19	0.128	0.141
5	5	0.5	0.7	30.333	1.858	1.821	15	30	0.2	0.5	0.186	0.13	0.142
5	5	0.6	0.2	15.691	1.594	1.555	15	30	0.2	0.7	0.189	0.13	0.142
5	5	0.6	0.5	171.096	1.616	1.592	15	30	0.5	0.2	0.166	0.23	0.236
5	5	0.6	0.7	122.525	1.71	1.69	15	30	0.5	0.5	0.157	0.231	0.238
5	10	0.2	0.2	20.815	0.758	0.761	15	30	0.5	0.7	0.155	0.231	0.237
5	10	0.2	0.5	36.747	0.844	0.835	15	30	0.6	0.2	0.153	0.261	0.266
5	10	0.2	0.7	38.392	0.883	0.878	15	30	0.6	0.5	0.142	0.262	0.267
5	10	0.5	0.2	8.447	0.699	0.697	15	30	0.6	0.7	0.135	0.263	0.268
5	10	0.5	0.5	13.505	0.713	0.705	15	40	0.2	0.2	0.161	0.122	0.131
5	10	0.5	0.7	12.165	0.799	0.796	15	40	0.2	0.5	0.16	0.125	0.134
5	10	0.6	0.2	15.714	0.763	0.75	15	40	0.2	0.7	0.16	0.125	0.134
5	10	0.6	0.5	6.207	0.675	0.674	15	40	0.5	0.2	0.14	0.22	0.227
5	10	0.6	0.7	22.794	0.74	0.728	15	40	0.5	0.5	0.135	0.219	0.225
5	20	0.2	0.2	1.301	0.325	0.344	15	40	0.5	0.7	0.129	0.219	0.225
5	20	0.2	0.5	0.905	0.315	0.343	15	40	0.6	0.2	0.129	0.252	0.258
5	20	0.2	0.7	4.667	0.371	0.386	15	40	0.6	0.5	0.12	0.251	0.256
5	20	0.5	0.2	6.983	0.368	0.374	15	40	0.6	0.7	0.117	0.253	0.258
5	20	0.5	0.5	0.504	0.366	0.37	30	5	0.2	0.2	2.041	0.705	0.706
5	20	0.5	0.7	0.866	0.367	0.376	30	5	0.2	0.5	2.276	0.707	0.708
5	20	0.6	0.2	2.347	0.39	0.396	30	5	0.2	0.7	57.25	0.727	0.727
5	20	0.6	0.5	0.58	0.365	0.37	30	5	0.5	0.2	2.991	0.579	0.579
5	20	0.6	0.7	2.782	0.368	0.372	30	5	0.5	0.5	4.882	0.583	0.584
5	30	0.2	0.2	1.821	0.176	0.201	30	5	0.5	0.7	5.315	0.658	0.66
5	30	0.2	0.5	0.34	0.184	0.21	30	5	0.6	0.2	2.366	0.54	0.54
5	30	0.2	0.7	0.337	0.192	0.216	30	5	0.6	0.5	1.13	0.542	0.543
5	30	0.5	0.2	0.346	0.282	0.288	30	5	0.6	0.7	126.33	0.525	0.525
5	30	0.5	0.5	0.618	0.277	0.283	30	10	0.2	0.2	0.422	0.165	0.199
5	30	0.5	0.7	0.284	0.281	0.287	30	10	0.2	0.5	0.366	0.184	0.218
5	30	0.6	0.2	0.861	0.309	0.315	30	10	0.2	0.7	0.347	0.16	0.198
5	30	0.6	0.5	2.374	0.307	0.314	30	10	0.5	0.2	0.309	0.294	0.299
5	30	0.6	0.7	0.316	0.302	0.308	30	10	0.5	0.5	0.322	0.29	0.295
5	40	0.2	0.2	0.248	0.145	0.164	30	10	0.5	0.7	0.541	0.3	0.305
5	40	0.2	0.5	0.239	0.143	0.165	30	10	0.6	0.2	0.273	0.324	0.328
5	40	0.2	0.7	0.283	0.144	0.166	30	10	0.6	0.5	1.181	0.327	0.331
5	40	0.5	0.2	0.54	0.249	0.255	30	10	0.6	0.7	0.253	0.331	0.336
5	40	0.5	0.5	0.232	0.257	0.264	30	20	0.2	0.2	0.218	0.133	0.149
5	40	0.5	0.7	0.196	0.254	0.261	30	20	0.2	0.5	0.214	0.134	0.149
5	40	0.6	0.2	0.222	0.279	0.286	30	20	0.2	0.7	0.211	0.134	0.15
5	40	0.6	0.5	0.195	0.28	0.287	30	20	0.5	0.2	0.186	0.243	0.249
5	40	0.6	0.7	0.175	0.282	0.289	30	20	0.5	0.5	0.178	0.243	0.249
15	5	0.2	0.2	12.929	0.902	0.898	30	20	0.5	0.7	0.174	0.246	0.253
15	5	0.2	0.5	24.547	0.906	0.913	30	20	0.6	0.2	0.165	0.277	0.282
15	5	0.2	0.7	23.651	0.925	0.926	30	20	0.6	0.5	0.156	0.276	0.281
15	5	0.5	0.2	5.145	0.802	0.8	30	20	0.6	0.7	0.156	0.278	0.283
15	5	0.5	0.5	33.086	0.776	0.776	30	30	0.2	0.2	0.172	0.126	0.135
15	5	0.5	0.7	11.948	0.795	0.794	30	30	0.2	0.5	0.171	0.126	0.135
15	5	0.6	0.2	15.276	0.732	0.731	30	30	0.2	0.7	0.168	0.126	0.135
15	5	0.6	0.5	3.845	0.742	0.74	30	30	0.5	0.2	0.146	0.228	0.234
15	5	0.6	0.7	12.678	0.736	0.737	30	30	0.5	0.5	0.143	0.226	0.232
15	10	0.2	0.2	1.994	0.291	0.315	30	30	0.5	0.7	0.14	0.227	0.233
15	10	0.2	0.5	0.616	0.284	0.313	30	30	0.6	0.2	0.129	0.259	0.263
15	10	0.2	0.7	0.95	0.287	0.314	30	30	0.6	0.5	0.123	0.26	0.265
15	10	0.5	0.2	1.122	0.345	0.351	30	30	0.6	0.7	0.12	0.26	0.264
15	10	0.5	0.5	0.693	0.342	0.348	30	40	0.2	0.2	0.146	0.12	0.126
15	10	0.5	0.7	1.563	0.34	0.345	30	40	0.2	0.5	0.144	0.122	0.128
15	10	0.6	0.2	13.285	0.367	0.373	30	40	0.2	0.7	0.143	0.12	0.126
15	10	0.6	0.5	2.519	0.36	0.366	30	40	0.5	0.2	0.123	0.215	0.221
15	10	0.6	0.7	1.467	0.361	0.365	30	40	0.5	0.5	0.118	0.215	0.221
15	20	0.2	0.2	0.245	0.135	0.156	30	40	0.5	0.7	0.118	0.217	0.223
15	20	0.2	0.5	0.344	0.139	0.161	30	40	0.6	0.2	0.111	0.251	0.254
15	20	0.2	0.7	0.245	0.141	0.163	30	40	0.6	0.5	0.105	0.25	0.253
15	20	0.5	0.2	0.213	0.251	0.257	30	40	0.6	0.7	0.101	0.251	0.255
15	20	0.5	0.5	0.206	0.254	0.259

**Table 5:** RMSE values of the ML (RMSE  ) and the Bayesian estimators (RMSE  represents the Bayesian with  and RMSE  represents the Bayesian with  ) for ICC  and different values of *n*, *J*,  , and

				RMSE	RMSE	RMSE					RMSE	RMSE	RMSE
5	5	0.2	0.2	25.163	1.146	1.126	15	20	0.5	0.7	0.122	0.161	0.175
5	5	0.2	0.5	10.82	1.242	1.221	15	20	0.6	0.2	0.1	0.17	0.177
5	5	0.2	0.7	1591.347	1.281	1.257	15	20	0.6	0.5	0.093	0.173	0.181
5	5	0.5	0.2	35.852	1.098	1.086	15	20	0.6	0.7	0.085	0.175	0.182
5	5	0.5	0.5	9.419	1.04	1.023	15	30	0.2	0.2	0.136	0.103	0.12
5	5	0.5	0.7	10.523	1.092	1.077	15	30	0.2	0.5	0.137	0.103	0.121
5	5	0.6	0.2	16.765	1.05	1.028	15	30	0.2	0.7	0.136	0.104	0.121
5	5	0.6	0.5	27.568	1.048	1.033	15	30	0.5	0.2	0.103	0.137	0.149
5	5	0.6	0.7	14.273	1.031	1.023	15	30	0.5	0.5	0.097	0.139	0.151
5	10	0.2	0.2	3.749	0.334	0.371	15	30	0.5	0.7	0.094	0.139	0.151
5	10	0.2	0.5	1.869	0.356	0.383	15	30	0.6	0.2	0.079	0.154	0.161
5	10	0.2	0.7	41.055	0.396	0.428	15	30	0.6	0.5	0.072	0.154	0.159
5	10	0.5	0.2	1.281	0.345	0.359	15	30	0.6	0.7	0.067	0.154	0.16
5	10	0.5	0.5	2.041	0.323	0.337	15	40	0.2	0.2	0.115	0.093	0.108
5	10	0.5	0.7	12.806	0.344	0.358	15	40	0.2	0.5	0.114	0.094	0.108
5	10	0.6	0.2	179.541	0.346	0.363	15	40	0.2	0.7	0.113	0.094	0.109
5	10	0.6	0.5	0.501	0.323	0.334	15	40	0.5	0.2	0.088	0.128	0.138
5	10	0.6	0.7	2.179	0.311	0.322	15	40	0.5	0.5	0.084	0.128	0.138
5	20	0.2	0.2	0.24	0.134	0.172	15	40	0.5	0.7	0.082	0.129	0.14
5	20	0.2	0.5	0.242	0.136	0.177	15	40	0.6	0.2	0.068	0.143	0.148
5	20	0.2	0.7	0.273	0.148	0.185	15	40	0.6	0.5	0.062	0.144	0.15
5	20	0.5	0.2	0.208	0.194	0.213	15	40	0.6	0.7	0.058	0.145	0.149
5	20	0.5	0.5	0.186	0.19	0.208	30	5	0.2	0.2	3.068	0.519	0.517
5	20	0.5	0.7	0.179	0.197	0.215	30	5	0.2	0.5	1.998	0.521	0.521
5	20	0.6	0.2	0.212	0.209	0.224	30	5	0.2	0.7	1.584	0.519	0.519
5	20	0.6	0.5	0.167	0.206	0.22	30	5	0.5	0.2	1.417	0.365	0.365
5	20	0.6	0.7	0.156	0.21	0.223	30	5	0.5	0.5	1.486	0.366	0.366
5	30	0.2	0.2	0.181	0.117	0.144	30	5	0.5	0.7	0.624	0.357	0.358
5	30	0.2	0.5	0.177	0.118	0.144	30	5	0.6	0.2	1.038	0.282	0.283
5	30	0.2	0.7	0.178	0.119	0.145	30	5	0.6	0.5	0.281	0.247	0.247
5	30	0.5	0.2	0.157	0.163	0.181	30	5	0.6	0.7	0.434	0.247	0.247
5	30	0.5	0.5	0.145	0.164	0.182	30	10	0.2	0.2	0.261	0.135	0.174
5	30	0.5	0.7	0.136	0.166	0.182	30	10	0.2	0.5	0.25	0.13	0.169
5	30	0.6	0.2	0.142	0.172	0.185	30	10	0.2	0.7	0.258	0.137	0.175
5	30	0.6	0.5	0.125	0.173	0.185	30	10	0.5	0.2	0.181	0.2	0.213
5	30	0.6	0.7	0.117	0.178	0.19	30	10	0.5	0.5	0.174	0.203	0.216
5	40	0.2	0.2	0.153	0.109	0.13	30	10	0.5	0.7	0.171	0.204	0.216
5	40	0.2	0.5	0.155	0.111	0.133	30	10	0.6	0.2	0.131	0.214	0.219
5	40	0.2	0.7	0.152	0.111	0.133	30	10	0.6	0.5	0.116	0.215	0.221
5	40	0.5	0.2	0.13	0.144	0.16	30	10	0.6	0.7	0.113	0.217	0.224
5	40	0.5	0.5	0.123	0.148	0.166	30	20	0.2	0.2	0.158	0.112	0.132
5	40	0.5	0.7	0.115	0.149	0.164	30	20	0.2	0.5	0.158	0.111	0.131
5	40	0.6	0.2	0.119	0.157	0.169	30	20	0.2	0.7	0.159	0.113	0.132
5	40	0.6	0.5	0.106	0.162	0.173	30	20	0.5	0.2	0.11	0.152	0.164
5	40	0.6	0.7	0.097	0.162	0.172	30	20	0.5	0.5	0.109	0.152	0.164
15	5	0.2	0.2	4.898	0.637	0.637	30	20	0.5	0.7	0.106	0.156	0.168
15	5	0.2	0.5	1.439	0.601	0.602	30	20	0.6	0.2	0.077	0.168	0.173
15	5	0.2	0.7	1.517	0.618	0.613	30	20	0.6	0.5	0.073	0.17	0.175
15	5	0.5	0.2	0.851	0.466	0.467	30	20	0.6	0.7	0.071	0.171	0.176
15	5	0.5	0.5	2.01	0.456	0.46	30	30	0.2	0.2	0.127	0.099	0.115
15	5	0.5	0.7	1.7	0.475	0.477	30	30	0.2	0.5	0.126	0.1	0.114
15	5	0.6	0.2	1.784	0.41	0.411	30	30	0.2	0.7	0.125	0.1	0.115
15	5	0.6	0.5	2.641	0.396	0.395	30	30	0.5	0.2	0.088	0.136	0.146
15	5	0.6	0.7	3.921	0.372	0.374	30	30	0.5	0.5	0.088	0.139	0.149
15	10	0.2	0.2	0.291	0.155	0.195	30	30	0.5	0.7	0.086	0.137	0.146
15	10	0.2	0.5	0.284	0.15	0.193	30	30	0.6	0.2	0.062	0.154	0.157
15	10	0.2	0.7	0.281	0.145	0.189	30	30	0.6	0.5	0.057	0.153	0.157
15	10	0.5	0.2	0.209	0.21	0.225	30	30	0.6	0.7	0.055	0.155	0.158
15	10	0.5	0.5	0.21	0.216	0.229	30	40	0.2	0.2	0.108	0.089	0.103
15	10	0.5	0.7	0.197	0.216	0.23	30	40	0.2	0.5	0.106	0.09	0.103
15	10	0.6	0.2	0.26	0.227	0.236	30	40	0.2	0.7	0.107	0.091	0.105
15	10	0.6	0.5	0.782	0.226	0.235	30	40	0.5	0.2	0.077	0.128	0.137
15	10	0.6	0.7	2.011	0.228	0.237	30	40	0.5	0.5	0.076	0.128	0.136
15	20	0.2	0.2	0.176	0.115	0.139	30	40	0.5	0.7	0.074	0.127	0.135
15	20	0.2	0.5	0.172	0.117	0.141	30	40	0.6	0.2	0.052	0.145	0.148
15	20	0.2	0.7	0.172	0.115	0.138	30	40	0.6	0.5	0.05	0.147	0.15
15	20	0.5	0.2	0.131	0.155	0.169	30	40	0.6	0.7	0.048	0.146	0.15
15	20	0.5	0.5	0.124	0.16	0.174

**Table 6:** Relative bias in % of the ML (Bias_ML_) and the Bayesian estimators (Bias_Bay_ represents the Bayesian with  and Bias_BML_ represents the Bayesian with  ) for ICC  and different values of *n*, *J*,  , and

				Bias	Bias	Bias					Bias	Bias	Bias
5	5	0.2	0.2	939.706	175.218	177.665	15	20	0.5	0.7	23.76	67.163	65.211
5	5	0.2	0.5	2576.35	344.055	340.826	15	20	0.6	0.2	102.766	32.639	31.891
5	5	0.2	0.7	1012.501	456.048	461.978	15	20	0.6	0.5	151.732	52.393	50.838
5	5	0.5	0.2	108.197	84.818	81.633	15	20	0.6	0.7	639.383	63.361	61.91
5	5	0.5	0.5	9.274	162.483	163.005	15	30	0.2	0.2	200.772	65.025	62.464
5	5	0.5	0.7	526.117	210.689	210.87	15	30	0.2	0.5	167.176	104.229	100.129
5	5	0.6	0.2	173.361	90.041	87.431	15	30	0.2	0.7	18.683	137.57	133.024
5	5	0.6	0.5	49.158	135.665	134.478	15	30	0.5	0.2	161.756	47.165	45.048
5	5	0.6	0.7	699.459	178.635	179.595	15	30	0.5	0.5	47.962	62.547	60.248
5	10	0.2	0.2	1064.461	128.863	127.003	15	30	0.5	0.7	75.302	76.234	73.687
5	10	0.2	0.5	203.735	207.413	208.761	15	30	0.6	0.2	67.323	43.167	41.534
5	10	0.2	0.7	94.878	297.133	296.062	15	30	0.6	0.5	19.332	59.943	58.139
5	10	0.5	0.2	93.408	77.668	76.457	15	30	0.6	0.7	17.13	63.18	61.173
5	10	0.5	0.5	654.471	102.57	103.688	15	40	0.2	0.2	36.969	72.628	69.546
5	10	0.5	0.7	332.931	144.312	146.785	15	40	0.2	0.5	15.178	101.716	98.562
5	10	0.6	0.2	169.053	59.41	59.602	15	40	0.2	0.7	103.122	122.674	119.056
5	10	0.6	0.5	63.505	109.252	108.353	15	40	0.5	0.2	54.952	56.233	53.458
5	10	0.6	0.7	1353.22	122.199	123.637	15	40	0.5	0.5	14.505	71.001	68.306
5	20	0.2	0.2	313.298	94.42	90.214	15	40	0.5	0.7	31.637	74.519	71.875
5	20	0.2	0.5	1435.496	175.674	172.445	15	40	0.6	0.2	3.904	55.525	53.487
5	20	0.2	0.7	104.246	207.484	206.478	15	40	0.6	0.5	15.277	66.395	64.375
5	20	0.5	0.2	1695.914	54.03	54.491	15	40	0.6	0.7	28.682	71.796	69.65
5	20	0.5	0.5	43.633	84.151	84.021	30	5	0.2	0.2	1394.99	57.697	57.462
5	20	0.5	0.7	321.686	101.773	101.497	30	5	0.2	0.5	6438.42	92.174	91.853
5	20	0.6	0.2	216.689	54.383	55.371	30	5	0.2	0.7	6188.805	107.343	107.263
5	20	0.6	0.5	51.553	75.3	74.366	30	5	0.5	0.2	177.108	33.585	32.785
5	20	0.6	0.7	26.749	96.432	96.959	30	5	0.5	0.5	128.246	48.822	49.195
5	30	0.2	0.2	21.324	87.631	87.895	30	5	0.5	0.7	37.675	63.07	63.138
5	30	0.2	0.5	83.263	133.207	128.895	30	5	0.6	0.2	192.766	26.28	26.152
5	30	0.2	0.7	1292.766	168.866	164.89	30	5	0.6	0.5	226.727	49.149	49.239
5	30	0.5	0.2	87.437	48.346	48.156	30	5	0.6	0.7	244.26	50.056	50.014
5	30	0.5	0.5	114.345	75.708	73.846	30	10	0.2	0.2	887.138	40.248	38.253
5	30	0.5	0.7	129.019	89.891	88.584	30	10	0.2	0.5	91.978	90.081	86.919
5	30	0.6	0.2	203.25	47.662	47.134	30	10	0.2	0.7	48.618	107.919	104.33
5	30	0.6	0.5	26.482	68.369	67.769	30	10	0.5	0.2	43.356	35.66	34.49
5	30	0.6	0.7	48.218	79.373	79.442	30	10	0.5	0.5	134.341	48.188	46.45
5	40	0.2	0.2	28.181	70.438	71.177	30	10	0.5	0.7	572.786	59.396	57.443
5	40	0.2	0.5	47.091	141.974	137.019	30	10	0.6	0.2	180.012	30.446	29.172
5	40	0.2	0.7	169.648	166.692	159.894	30	10	0.6	0.5	262.174	42.491	41.197
5	40	0.5	0.2	232.466	41.92	41.522	30	10	0.6	0.7	221.584	49.66	48.066
5	40	0.5	0.5	50.815	71.833	70.561	30	20	0.2	0.2	17.57	70.202	68.437
5	40	0.5	0.7	65.781	88.633	87.188	30	20	0.2	0.5	76.648	87.522	83.764
5	40	0.6	0.2	95.755	41.248	41.131	30	20	0.2	0.7	102.031	105.512	102.991
5	40	0.6	0.5	337.021	62.363	61.601	30	20	0.5	0.2	25.786	64.208	61.509
5	40	0.6	0.7	137.401	75.084	73.927	30	20	0.5	0.5	195.755	68.995	65.967
15	5	0.2	0.2	1382.23	66.236	65.608	30	20	0.5	0.7	12.808	75.91	72.546
15	5	0.2	0.5	3114.31	123.3	124.703	30	20	0.6	0.2	36.709	60.133	57.464
15	5	0.2	0.7	762.222	180.02	177.251	30	20	0.6	0.5	28.893	68.769	66.26
15	5	0.5	0.2	312.494	45.271	45.203	30	20	0.6	0.7	8.648	74.745	72.346
15	5	0.5	0.5	158.832	73.883	73.922	30	30	0.2	0.2	16.632	83.612	80.43
15	5	0.5	0.7	176.129	89.463	88.718	30	30	0.2	0.5	4.256	84.067	81.135
15	5	0.6	0.2	47.277	48.753	50.276	30	30	0.2	0.7	22.266	88.912	85.758
15	5	0.6	0.5	285.822	61.179	61.678	30	30	0.5	0.2	12.282	75.513	72.03
15	5	0.6	0.7	40.838	83.226	82.861	30	30	0.5	0.5	12.309	79.911	76.956
15	10	0.2	0.2	1266.762	41.67	41.652	30	30	0.5	0.7	2.186	80.325	77.784
15	10	0.2	0.5	21970.6	106.428	104.369	30	30	0.6	0.2	9.385	74.856	71.836
15	10	0.2	0.7	330.852	127.641	125.988	30	30	0.6	0.5	1.032	78.497	75.826
15	10	0.5	0.2	61.867	30.545	30.122	30	30	0.6	0.7	8.66	80.323	77.907
15	10	0.5	0.5	108.936	50.674	49.646	30	40	0.2	0.2	1.012	83.597	79.347
15	10	0.5	0.7	55.614	64.946	64.667	30	40	0.2	0.5	35.467	86.099	83.287
15	10	0.6	0.2	311.448	32.714	32.54	30	40	0.2	0.7	22.23	89.364	86.915
15	10	0.6	0.5	19.852	49.811	49.947	30	40	0.5	0.2	2.428	80.059	76.881
15	10	0.6	0.7	67.793	60.111	59.945	30	40	0.5	0.5	0.828	82.002	79.127
15	20	0.2	0.2	354.039	57.176	54.656	30	40	0.5	0.7	1.092	81.837	79.248
15	20	0.2	0.5	1217.28	109.289	105.68	30	40	0.6	0.2	2.463	78.273	75.699
15	20	0.2	0.7	163.248	142.24	136.652	30	40	0.6	0.5	1.436	79.898	77.626
15	20	0.5	0.2	61.296	38.271	36.857	30	40	0.6	0.7	1.31	80.875	78.575
15	20	0.5	0.5	205.614	57.65	55.776

**Table 7:** Relative bias in % of the ML (Bias  ) and the Bayesian estimators (Bias  ) represents the Bayesian with  and Bias  represents the Bayesian with  ) for ICC  and different values of *n*, *J*,  , and

				Bias	Bias	Bias					Bias	Bias	Bias
5	5	0.2	0.2	180.425	97.729	97.848	15	20	0.5	0.7	1.779	68.523	65.365
5	5	0.2	0.5	4085.79	296.848	296.841	15	20	0.6	0.2	44.621	55.605	52.813
5	5	0.2	0.7	1867.08	353.484	349.919	15	20	0.6	0.5	3.034	60.238	57.617
5	5	0.5	0.2	50.618	49.194	50.365	15	20	0.6	0.7	5.119	64.958	62.425
5	5	0.5	0.5	112.359	99.353	100.261	15	30	0.2	0.2	6.011	74.512	69.163
5	5	0.5	0.7	20.548	149.938	151.061	15	30	0.2	0.5	14.988	81.933	77.68
5	5	0.6	0.2	20.156	42.548	41.06	15	30	0.2	0.7	21.005	88.347	85.029
5	5	0.6	0.5	738.784	86.14	86.637	15	30	0.5	0.2	2.785	68.067	64.702
5	5	0.6	0.7	209.891	137.535	137.512	15	30	0.5	0.5	7.305	70.079	66.955
5	10	0.2	0.2	702.919	82.189	81.101	15	30	0.5	0.7	6.319	72.935	70.103
5	10	0.2	0.5	209.136	182.931	184.313	15	30	0.6	0.2	8.127	65.341	62.491
5	10	0.2	0.7	396.677	250.645	246.332	15	30	0.6	0.5	5.803	67.464	65.019
5	10	0.5	0.2	213.951	31.923	32.773	15	30	0.6	0.7	1.144	69.318	66.917
5	10	0.5	0.5	608.719	70.512	72.082	15	40	0.2	0.2	1.56	76.63	71.004
5	10	0.5	0.7	292.342	107.28	106.708	15	40	0.2	0.5	27.766	80.567	76.371
5	10	0.6	0.2	114.348	31.499	33.501	15	40	0.2	0.7	132.798	82.327	77.86
5	10	0.6	0.5	37.868	60.978	60.269	15	40	0.5	0.2	6.269	70.345	67.259
5	10	0.6	0.7	322.257	86.07	84.107	15	40	0.5	0.5	0.227	71.222	68.422
5	20	0.2	0.2	5140.523	74.28	73.683	15	40	0.5	0.7	2.364	72.428	69.788
5	20	0.2	0.5	738.356	136.74	131.187	15	40	0.6	0.2	3.306	68.78	66.52
5	20	0.2	0.7	973.423	193.073	183.85	15	40	0.6	0.5	0.237	70.115	68.227
5	20	0.5	0.2	33.357	26.839	26.188	15	40	0.6	0.7	1.657	70.571	68.814
5	20	0.5	0.5	29.743	56.85	55.812	30	5	0.2	0.2	71.264	30.015	30.151
5	20	0.5	0.7	293.616	80.078	78.315	30	5	0.2	0.5	203.093	47.948	48.552
5	20	0.6	0.2	3064.897	23.056	22.691	30	5	0.2	0.7	162.961	57.815	57.962
5	20	0.6	0.5	141.048	49.363	48.477	30	5	0.5	0.2	65.295	11.699	11.46
5	20	0.6	0.7	183.525	64.047	63.43	30	5	0.5	0.5	33.614	27.163	27.075
5	30	0.2	0.2	63.151	54.592	50.382	30	5	0.5	0.7	52.018	27.074	27.23
5	30	0.2	0.5	88.897	115.98	108.684	30	5	0.6	0.2	296.281	7.696	7.805
5	30	0.2	0.7	74.222	156.856	147.408	30	5	0.6	0.5	20.843	17.36	17.523
5	30	0.5	0.2	5.832	28.231	27.638	30	5	0.6	0.7	67.838	27.974	27.849
5	30	0.5	0.5	90.982	51.166	49.514	30	10	0.2	0.2	15.486	56.606	52.52
5	30	0.5	0.7	56.225	65.307	63.523	30	10	0.2	0.5	25.161	77.947	74.029
5	30	0.6	0.2	25.728	26.956	26.756	30	10	0.2	0.7	287.394	97.207	92.745
5	30	0.6	0.5	76.56	47.217	46.01	30	10	0.5	0.2	23.314	49.55	45.933
5	30	0.6	0.7	18.263	57.962	56.192	30	10	0.5	0.5	64.459	55.552	52.107
5	40	0.2	0.2	59.795	57.455	52.962	30	10	0.5	0.7	4.539	62.899	59.47
5	40	0.2	0.5	104.315	101.762	94.837	30	10	0.6	0.2	20.208	48.932	46.516
5	40	0.2	0.7	2423.67	146.467	137.357	30	10	0.6	0.5	23.429	57.004	54.44
5	40	0.5	0.2	71.194	34.808	33.115	30	10	0.6	0.7	20.733	61.414	58.784
5	40	0.5	0.5	17.511	56.792	54.364	30	20	0.2	0.2	3.856	77.33	71.309
5	40	0.5	0.7	9.612	68.39	66.062	30	20	0.2	0.5	9.562	79.292	74.84
5	40	0.6	0.2	42.379	30.542	29.469	30	20	0.2	0.7	16.742	82.423	78.538
5	40	0.6	0.5	7.197	48.516	46.937	30	20	0.5	0.2	0.587	72.727	69.369
5	40	0.6	0.7	80.748	59.465	57.386	30	20	0.5	0.5	0.296	73.087	69.506
15	5	0.2	0.2	490.349	40.138	39.193	30	20	0.5	0.7	1.527	73.836	70.474
15	5	0.2	0.5	2803.13	93.016	95.135	30	20	0.6	0.2	40.58	70.874	68.019
15	5	0.2	0.7	94231.7	118.795	118.882	30	20	0.6	0.5	2.45	71.272	68.618
15	5	0.5	0.2	446.312	20.598	20.48	30	20	0.6	0.7	0.147	72.023	69.408
15	5	0.5	0.5	8.249	39.589	39.418	30	30	0.2	0.2	3.493	77.961	72.732
15	5	0.5	0.7	686.354	50.31	50.278	30	30	0.2	0.5	1.877	77.558	72.005
15	5	0.6	0.2	472.988	14.685	15.161	30	30	0.2	0.7	6.558	78.139	73.528
15	5	0.6	0.5	187.689	26.937	27.048	30	30	0.5	0.2	1.787	72.357	69.404
15	5	0.6	0.7	27.892	51.789	51.997	30	30	0.5	0.5	1.615	72.959	70.207
15	10	0.2	0.2	230.013	35.213	31.963	30	30	0.5	0.7	2.106	72.922	70.443
15	10	0.2	0.5	344.476	82.708	78.286	30	30	0.6	0.2	3.399	70.612	68.31
15	10	0.2	0.7	15.321	111.873	106.684	30	30	0.6	0.5	0.584	70.95	68.985
15	10	0.5	0.2	22.65	28.692	27.291	30	30	0.6	0.7	0.895	71.066	69.293
15	10	0.5	0.5	64.502	41.437	39.689	30	40	0.2	0.2	3.362	77.598	72.447
15	10	0.5	0.7	2.906	54.824	52.782	30	40	0.2	0.5	0.231	76.673	71.081
15	10	0.6	0.2	39.196	23.171	22.183	30	40	0.2	0.7	7.209	77.56	72.785
15	10	0.6	0.5	44.949	39.477	38.189	30	40	0.5	0.2	2.468	70.765	68.271
15	10	0.6	0.7	32.648	43.632	41.748	30	40	0.5	0.5	0.038	70.983	68.802
15	20	0.2	0.2	39.603	64.419	58.788	30	40	0.5	0.7	0.553	71.21	69.095
15	20	0.2	0.5	114.491	90.242	86.207	30	40	0.6	0.2	0.738	69.428	68.01
15	20	0.2	0.7	81.633	97.36	92.204	30	40	0.6	0.5	1.48	69.411	67.777
15	20	0.5	0.2	11.16	57.031	53.31	30	40	0.6	0.7	0.532	69.658	68.31
15	20	0.5	0.5	4.442	63.884	60.681

**Table 8:** Relative bias in % of the ML (Bias  ) and the Bayesian estimators (Bias  represents the Bayesian with  and Bias_BML_ represents the Bayesian with  ) for ICC  and different values of *n*, *J*,  , and

				Bias	Bias	Bias					Bias	Bias	Bias
5	5	0.2	0.2	380.903	7.31	5.4	15	20	0.5	0.7	0.998	46.918	45.141
5	5	0.2	0.5	297.1	95.193	90.933	15	20	0.6	0.2	1.699	44.539	43.227
5	5	0.2	0.7	150.267	143.281	141.121	15	20	0.6	0.5	0.883	44.362	43.218
5	5	0.5	0.2	56.277	46.127	44.998	15	20	0.6	0.7	0.51	44.749	43.928
5	5	0.5	0.5	432.617	0.206	0.453	15	30	0.2	0.2	1.117	56.168	47.821
5	5	0.5	0.7	61.206	27.888	27.918	15	30	0.2	0.5	2.359	57.306	50.176
5	5	0.6	0.2	13.767	43.107	40.977	15	30	0.2	0.7	2.578	56.83	49.285
5	5	0.6	0.5	485.798	10.824	10.598	15	30	0.5	0.2	0.554	43.555	42.309
5	5	0.6	0.7	247.168	1.55	1.634	15	30	0.5	0.5	0.128	43.808	42.703
5	10	0.2	0.2	131.468	35.023	27.847	15	30	0.5	0.7	0.43	43.752	42.498
5	10	0.2	0.5	185.621	89.478	77.998	15	30	0.6	0.2	1.457	41.903	41.005
5	10	0.2	0.7	248.119	108.161	97.15	15	30	0.6	0.5	0.287	41.945	41.364
5	10	0.5	0.2	31.098	11.137	8.976	15	30	0.6	0.7	0.033	42.031	41.507
5	10	0.5	0.5	26.568	27.84	25.328	15	40	0.2	0.2	1.409	54.164	46.953
5	10	0.5	0.7	50.187	40.606	37.555	15	40	0.2	0.5	3.353	55.342	49.136
5	10	0.6	0.2	50.377	4.481	4.542	15	40	0.2	0.7	4.992	55.617	49.763
5	10	0.6	0.5	25.482	23.963	22.808	15	40	0.5	0.2	0.053	42.134	41.392
5	10	0.6	0.7	25.378	35.246	33.298	15	40	0.5	0.5	0.659	42.045	41.048
5	20	0.2	0.2	10.828	51.717	41.437	15	40	0.5	0.7	0.279	42.07	41.399
5	20	0.2	0.5	21.449	68.64	61.552	15	40	0.6	0.2	0.027	40.787	40.45
5	20	0.2	0.7	63.852	83.751	76.923	15	40	0.6	0.5	0.108	40.644	40.275
5	20	0.5	0.2	8.852	40.682	36.675	15	40	0.6	0.7	0.034	40.829	40.452
5	20	0.5	0.5	0.077	46.392	43.033	30	5	0.2	0.2	4.067	0.005	0
5	20	0.5	0.7	4.033	50.636	47.69	30	5	0.2	0.5	6.026	8.355	8.332
5	20	0.6	0.2	7.13	37.517	35.116	30	5	0.2	0.7	381.519	18.351	18.252
5	20	0.6	0.5	2.671	41.089	38.967	30	5	0.5	0.2	4.964	5.906	5.869
5	20	0.6	0.7	11.56	45.502	43.638	30	5	0.5	0.5	7.468	3.082	3.014
5	30	0.2	0.2	13.341	59.329	49.672	30	5	0.5	0.7	15.179	0.386	0.457
5	30	0.2	0.5	17.994	64.693	58.182	30	5	0.6	0.2	4.802	8.471	8.463
5	30	0.2	0.7	18	65.289	58.525	30	5	0.6	0.5	3.508	4.313	4.291
5	30	0.5	0.2	5.034	45.253	41.916	30	5	0.6	0.7	289.118	2.066	2.117
5	30	0.5	0.5	1.161	47.406	44.776	30	10	0.2	0.2	4.031	60.499	50.403
5	30	0.5	0.7	3.027	48.702	46.545	30	10	0.2	0.5	5.741	64.16	56.034
5	30	0.6	0.2	2.359	42.974	41.05	30	10	0.2	0.7	6.347	62.99	55.608
5	30	0.6	0.5	4.503	44.644	43.32	30	10	0.5	0.2	1.843	51.672	48.263
5	30	0.6	0.7	1.451	45.244	43.908	30	10	0.5	0.5	1.684	51.568	48.275
5	40	0.2	0.2	0.984	58.17	49.658	30	10	0.5	0.7	2.506	53.068	50.129
5	40	0.2	0.5	7.897	59.238	51.948	30	10	0.6	0.2	1.695	49.025	47.103
5	40	0.2	0.7	9.097	60.041	53.542	30	10	0.6	0.5	3.728	49.251	47.491
5	40	0.5	0.2	1.26	45.072	42.743	30	10	0.6	0.7	0.072	49.626	48.056
5	40	0.5	0.5	0.797	46.11	44.267	30	20	0.2	0.2	1.105	58.467	49.686
5	40	0.5	0.7	1.807	46.459	44.731	30	20	0.2	0.5	1.151	58.733	50.616
5	40	0.6	0.2	2.136	43.252	41.836	30	20	0.2	0.7	3.356	59.335	51.794
5	40	0.6	0.5	0.334	43.83	42.83	30	20	0.5	0.2	1.165	45.931	44.21
5	40	0.6	0.7	1.045	44.088	43.199	30	20	0.5	0.5	0.869	45.997	44.415
15	5	0.2	0.2	74.931	4.84	5.798	30	20	0.5	0.7	0.619	46.582	45.336
15	5	0.2	0.5	323.87	15.23	15.118	30	20	0.6	0.2	0.58	44.207	43.462
15	5	0.2	0.7	59.503	40.264	40.218	30	20	0.6	0.5	0.216	44.112	43.436
15	5	0.5	0.2	7.849	15.234	15.098	30	20	0.6	0.7	0.147	44.297	43.668
15	5	0.5	0.5	120.525	6.303	6.248	30	30	0.2	0.2	0.07	56.129	48.75
15	5	0.5	0.7	12.943	2.154	2.174	30	30	0.2	0.5	0.044	56.139	48.759
15	5	0.6	0.2	52.143	18.306	18.276	30	30	0.2	0.7	0.794	55.821	48.806
15	5	0.6	0.5	8.054	9.334	9.369	30	30	0.5	0.2	0.122	43.627	42.777
15	5	0.6	0.7	2.296	3.754	3.712	30	30	0.5	0.5	0.392	43.329	42.388
15	10	0.2	0.2	4.432	59.604	51.105	30	30	0.5	0.7	0.284	43.495	42.701
15	10	0.2	0.5	9.53	63.083	55.401	30	30	0.6	0.2	0.448	41.879	41.398
15	10	0.2	0.7	22.137	68.075	60.897	30	30	0.6	0.5	0.18	42.106	41.816
15	10	0.5	0.2	8.999	46.317	42.314	30	30	0.6	0.7	0.22	42.095	41.672
15	10	0.5	0.5	0.416	49.214	45.6	30	40	0.2	0.2	0.946	53.592	47.014
15	10	0.5	0.7	2.784	51.402	48.068	30	40	0.2	0.5	2.535	54.666	48.894
15	10	0.6	0.2	33.381	44.3	42.08	30	40	0.2	0.7	0.731	53.616	47.618
15	10	0.6	0.5	6.384	45.365	43.426	30	40	0.5	0.2	0.3	41.79	41.174
15	10	0.6	0.7	6.388	47.913	45.969	30	40	0.5	0.5	0.022	41.822	41.29
15	20	0.2	0.2	2.87	58.865	49.359	30	40	0.5	0.7	0.688	42.035	41.65
15	20	0.2	0.5	3.719	58.974	50.509	30	40	0.6	0.2	0.717	40.913	40.456
15	20	0.2	0.7	6.431	60.174	52.351	30	40	0.6	0.5	0.548	40.653	40.245
15	20	0.5	0.2	0.911	46.634	44.514	30	40	0.6	0.7	0.013	40.976	40.722
15	20	0.5	0.5	0.735	46.989	44.938

**Table 9:** Relative bias in % of the ML (Bias  ) and the Bayesian estimators (Bias  represents the Bayesian with  and Bias  represents the Bayesian with  ) for ICC  and different values of *n*, *J*,  , and

				Bias	Bias	Bias					Bias	Bias	Bias
5	5	0.2	0.2	164.985	30.853	30.064	15	20	0.5	0.7	0.22	27.013	26.284
5	5	0.2	0.5	91.398	2.688	0.009	15	20	0.6	0.2	0.573	24.739	24.365
5	5	0.2	0.7	11249.1	39.249	35.45	15	20	0.6	0.5	0.168	24.833	24.536
5	5	0.5	0.2	96.408	56.089	56.06	15	20	0.6	0.7	0.221	25.053	24.896
5	5	0.5	0.5	22.581	36.746	36.707	15	30	0.2	0.2	0.123	38.818	31.516
5	5	0.5	0.7	18.976	28.04	28.119	15	30	0.2	0.5	0.124	38.316	31.104
5	5	0.6	0.2	45.493	54.128	53.045	15	30	0.2	0.7	1.024	38.757	31.716
5	5	0.6	0.5	85.638	38.771	37.987	15	30	0.5	0.2	0.389	23.985	23.405
5	5	0.6	0.7	34.784	31.698	32.092	15	30	0.5	0.5	0.225	24.225	23.839
5	10	0.2	0.2	3.087	44.882	34.243	15	30	0.5	0.7	0.651	24.334	24.132
5	10	0.2	0.5	24.56	54.504	45.425	15	30	0.6	0.2	0.225	22.859	22.637
5	10	0.2	0.7	264.16	65.759	56.246	15	30	0.6	0.5	0.318	22.779	22.525
5	10	0.5	0.2	4.413	25.214	21.39	15	30	0.6	0.7	0.038	22.842	22.671
5	10	0.5	0.5	1.164	30.273	27.212	15	40	0.2	0.2	0.72	35.116	29.072
5	10	0.5	0.7	25.381	35.121	32.221	15	40	0.2	0.5	0.026	35.599	29.691
5	10	0.6	0.2	431.493	22.177	20.11	15	40	0.2	0.7	0.6	35.434	29.604
5	10	0.6	0.5	0.409	26.339	24.864	15	40	0.5	0.2	0.182	22.687	22.291
5	10	0.6	0.7	5.418	29.331	27.934	15	40	0.5	0.5	0.056	22.669	22.337
5	20	0.2	0.2	2.174	45.461	33.726	15	40	0.5	0.7	0.256	22.893	22.621
5	20	0.2	0.5	5.226	47.175	37.32	15	40	0.6	0.2	0.251	21.605	21.404
5	20	0.2	0.7	13.597	50.043	42.321	15	40	0.6	0.5	0.315	21.726	21.707
5	20	0.5	0.2	2.893	29.868	27.276	15	40	0.6	0.7	0.027	21.818	21.687
5	20	0.5	0.5	0.702	30.204	28.086	30	5	0.2	0.2	32.27	12.072	12.191
5	20	0.5	0.7	1.059	31.021	29.332	30	5	0.2	0.5	13.866	2.04	2.062
5	20	0.6	0.2	2.823	27.324	25.747	30	5	0.2	0.7	2.588	3.011	2.939
5	20	0.6	0.5	0.358	27.984	27.006	30	5	0.5	0.2	6.004	8.522	8.553
5	20	0.6	0.7	0.622	28.591	27.808	30	5	0.5	0.5	1.864	6.028	6.046
5	30	0.2	0.2	1.118	43.94	34.55	30	5	0.5	0.7	2.62	3.931	3.91
5	30	0.2	0.5	3.38	43.802	35.125	30	5	0.6	0.2	7.629	7.837	7.829
5	30	0.2	0.7	5.405	44.419	36.288	30	5	0.6	0.5	0.18	7.132	7.124
5	30	0.5	0.2	1.063	27.096	25.587	30	5	0.6	0.7	1.126	6.588	6.579
5	30	0.5	0.5	0.26	27.139	26.052	30	10	0.2	0.2	1.672	50.254	40.083
5	30	0.5	0.7	0.879	27.124	26.169	30	10	0.2	0.5	2.743	49.396	39.691
5	30	0.6	0.2	1.631	24.597	23.649	30	10	0.2	0.7	3.732	50.118	40.716
5	30	0.6	0.5	0.801	24.669	23.933	30	10	0.5	0.2	0.676	33.464	31.932
5	30	0.6	0.7	0.025	25.092	24.539	30	10	0.5	0.5	0.757	33.783	32.615
5	40	0.2	0.2	0.286	40.655	32.185	30	10	0.5	0.7	0.746	33.947	32.733
5	40	0.2	0.5	3.114	41.373	33.552	30	10	0.6	0.2	1.361	30.333	29.705
5	40	0.2	0.7	4.552	41.592	34.315	30	10	0.6	0.5	0.315	30.791	30.359
5	40	0.5	0.2	1.022	24.608	23.518	30	10	0.6	0.7	0.227	31.065	30.802
5	40	0.5	0.5	0.904	25.201	24.685	30	20	0.2	0.2	0.194	42.537	34.137
5	40	0.5	0.7	0.611	25.064	24.452	30	20	0.2	0.5	0.387	42.302	33.956
5	40	0.6	0.2	1.223	23.036	22.307	30	20	0.2	0.7	0.726	43.307	34.829
5	40	0.6	0.5	0.086	23.63	23.293	30	20	0.5	0.2	0.087	26.456	25.927
5	40	0.6	0.7	0.067	23.325	22.934	30	20	0.5	0.5	0.069	26.524	25.956
15	5	0.2	0.2	49.017	5.309	5.33	30	20	0.5	0.7	0.521	27.079	26.677
15	5	0.2	0.5	9.856	5.713	5.599	30	20	0.6	0.2	0.183	25.035	24.861
15	5	0.2	0.7	10.242	4.097	3.765	30	20	0.6	0.5	0.256	25.187	25.141
15	5	0.5	0.2	3.634	16.041	16.119	30	20	0.6	0.7	0.1	25.282	25.079
15	5	0.5	0.5	1.772	11.103	10.99	30	30	0.2	0.2	0.905	37.67	30.916
15	5	0.5	0.7	10.997	8.54	8.541	30	30	0.2	0.5	0.626	38.138	31.72
15	5	0.6	0.2	9.453	15.968	16.075	30	30	0.2	0.7	1.574	38.38	32.473
15	5	0.6	0.5	5.909	11.696	11.82	30	30	0.5	0.2	0.045	24.436	24.089
15	5	0.6	0.7	9.654	9.399	9.34	30	30	0.5	0.5	0.509	24.798	24.617
15	10	0.2	0.2	2.597	48.549	36.968	30	30	0.5	0.7	0.194	24.503	24.226
15	10	0.2	0.5	6.183	50.589	41.012	30	30	0.6	0.2	0.457	23.322	23.08
15	10	0.2	0.7	3.402	49.337	39.467	30	30	0.6	0.5	0.064	23.224	23.114
15	10	0.5	0.2	2.188	32.991	30.545	30	30	0.6	0.7	0.076	23.545	23.49
15	10	0.5	0.5	0.793	33.708	31.654	30	40	0.2	0.2	1.383	34.375	28.764
15	10	0.5	0.7	0.025	34.526	32.589	30	40	0.2	0.5	0.421	35.044	30.064
15	10	0.6	0.2	1.822	30.649	29.834	30	40	0.2	0.7	1.567	35.718	31.128
15	10	0.6	0.5	1.872	31.295	30.632	30	40	0.5	0.2	0.076	23.337	23.095
15	10	0.6	0.7	4.392	31.398	30.911	30	40	0.5	0.5	0.053	23.412	23.18
15	20	0.2	0.2	0.533	42.985	33.471	30	40	0.5	0.7	0.066	23.021	22.781
15	20	0.2	0.5	3.369	44.04	35.625	30	40	0.6	0.2	0.231	22.45	22.297
15	20	0.2	0.7	0.616	42.699	33.427	30	40	0.6	0.5	0.028	22.813	22.734
15	20	0.5	0.2	0.972	26.057	24.997	30	40	0.6	0.7	0.202	22.456	22.436
15	20	0.5	0.5	0.437	26.877	26.243							
